# Assessment of Corrosion in Naval Steels Submerged in Artificial Seawater Utilizing a Magnetic Non-Destructive Sensor

**DOI:** 10.3390/s25165015

**Published:** 2025-08-13

**Authors:** Polyxeni Vourna, Aphrodite Ktena, Evangelos V. Hristoforou, Nikolaos D. Papadopoulos

**Affiliations:** 1 Institute of Nanoscience and Nanotechnology, National Centre for Scientific Research “Demokritos”, 15341 Agia Paraskevi, Greece; 2 General Department, National and Kapodistrian University of Athens, 15784 Athens, Greece; apktena@uoa.gr; 3 Institute of Communication and Computer Systems, 15773 Athens, Greece; hristoforou@ece.ntua.gr; 4 Department of Research and Development, BFP Advanced Technologies G.P., 11633 Athens, Greece; npapadopoulos@bfp-tech.com

**Keywords:** naval steel, artificial seawater, corrosion products, electrochemical impedance spectroscopy, magnetic permeability, non-destructive evaluation, corrosion rate, DH36 steel, marine environment

## Abstract

This work presents a comprehensive evaluation of corrosion progression in DH36 naval steel through the integration of electrochemical impedance spectroscopy (EIS), weight loss, scanning electron microscopy (SEM), and advanced magnetic non-destructive techniques under artificial seawater (ASW, ASTM D1141) and natural marine conditions. Quantitative correlations are established between corrosion layer growth, electrochemical parameters, and magnetic permeability, demonstrating the magnetic sensor’s capacity for the real-time, non-invasive assessment of marine steel degradation. Laboratory exposures reveal a rapid initial corrosion phase with the formation of lepidocrocite and goethite, followed by the densification of the corrosion product layer and a pronounced decline in corrosion rate, ultimately governed by diffusion-controlled kinetics. Notably, changes in magnetic permeability closely track both the thickening of non-magnetic corrosion products and microstructural deterioration, with declining μmax and increased hysteresis widths (FWHM) sensitively indicating evolving surface conditions. A direct comparison with in situ marine immersion at Rafina confirms that the evolution of corrosion morphology and the corresponding magnetic response are further modulated by biofilm development, which exacerbates the attenuation of measured surface permeability and introduces greater variability linked to biological activity. These findings underscore the robustness and diagnostic potential of magnetic non-destructive sensors for the predictive, condition-based monitoring of naval steels, bridging laboratory-controlled observations and complex real-world environments with high quantitative fidelity to corrosion kinetics, phase evolution, and microstructural transformations, thus guiding the strategic deployment of protection and maintenance regimens for naval fleet integrity.

## 1. Introduction

Naval steels are extensively used in maritime structures due to their high strength and toughness [1]. However, continuous exposure to aggressive marine environments, characterized by high chloride content, oxygen availability, and fluctuating temperatures, accelerates corrosion [2]. Corrosion on ship hulls compromises structural integrity and safety issues [3], leading to safety hazards and expensive repairs. To combat this issue, various methods of corrosion prevention and mitigation (coatings, cathodic protection, corrosion-resistant alloys, and environmental control) have been developed to prolong the operational lifespan of marine steels and reduce maintenance costs [4].

A comparative analysis of the main corrosion protection methods used for naval steels is summarized in Table 1 and this is followed by a brief discussion of strengths, limitations, and typical use cases.

Protective coatings and galvanizing serve as primary barriers against corrosive agents, while cathodic protection provides continuous electrochemical safeguarding for submerged or buried structures. Alternative strategies, such as powder coating, metallizing, and petrolatum tape systems, offer tailored solutions for specific joint or component geometries, often as part of multilayered systems. Furthermore, the use of corrosion-resistant steel alloys is highlighted, although economic considerations may limit widespread adoption.

However, the early detection of corrosion in naval steels is essential for maintaining the structural integrity of maritime vessels and offshore installations. Given the harsh marine environment and the high cost of failures, both periodic inspections and continuous monitoring systems are employed to detect corrosion at its earliest stages [15]. Table 2 presents the characteristics of corrosion detection methods for steel.

A multifaceted approach is frequently employed for efficient corrosion detection in naval steels. Although optical and ultrasonic techniques are still viable for regular inspections, magnetic and electrochemical technologies are progressively preferred for early detection and real-time monitoring. The incorporation of intelligent sensing technology will augment predictive maintenance and the operational preparedness of naval fleets [18,21,22].

This study seeks to examine the corrosion of naval steels by analyzing the variations in the magnetic loop parameters of magnetic permeability. This research used a laboratory-based, stepwise electrochemical corrosion technique on naval steel samples immersed in simulated seawater to produce differing levels of corrosion. The samples are next scanned with a magnetic non-destructive sensor that captures the tangential component of the generated electrical signal. This signal relates to the magnetic permeability of naval steel. To evaluate the effectiveness of corrosion assessment on naval steel samples, the samples were submerged for different durations in the marine environment of Greece and later evaluated for corrosion levels using a magnetic sensor. The magnetic results were compared with those obtained using destructive corrosion characterization methods.

## 2. Materials and Methods

### 2.1. Material

**Figure 1:**
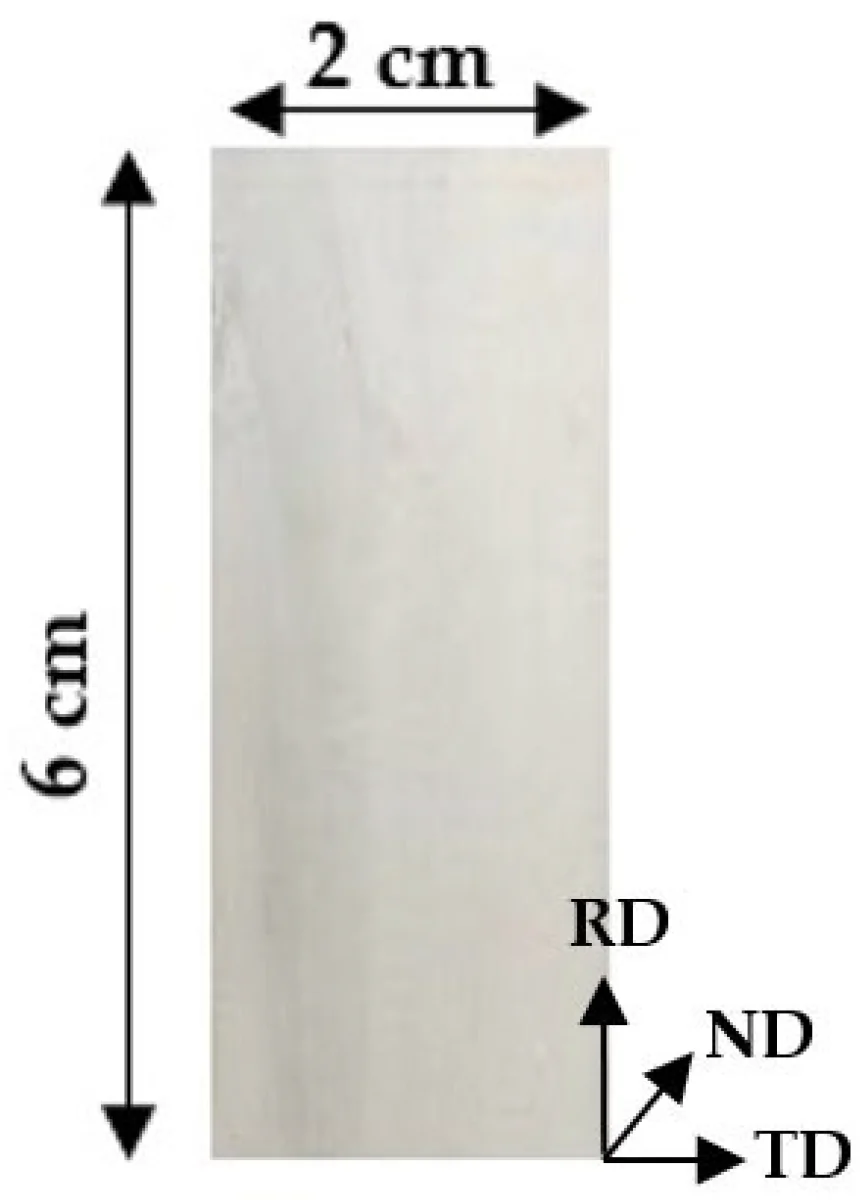
Dimensions of the as-received naval steel samples used.

A typical naval steel sample (DH36) served as the study alloy. Table 3 details the chemical composition of the sample as it was received. The dimensions of the sample are shown in Figure 1. The thickness of the material was 2 mm and its initial weight was (1884 ± 15) g.

### 2.2. Experimental Procedure

**Figure 2:**
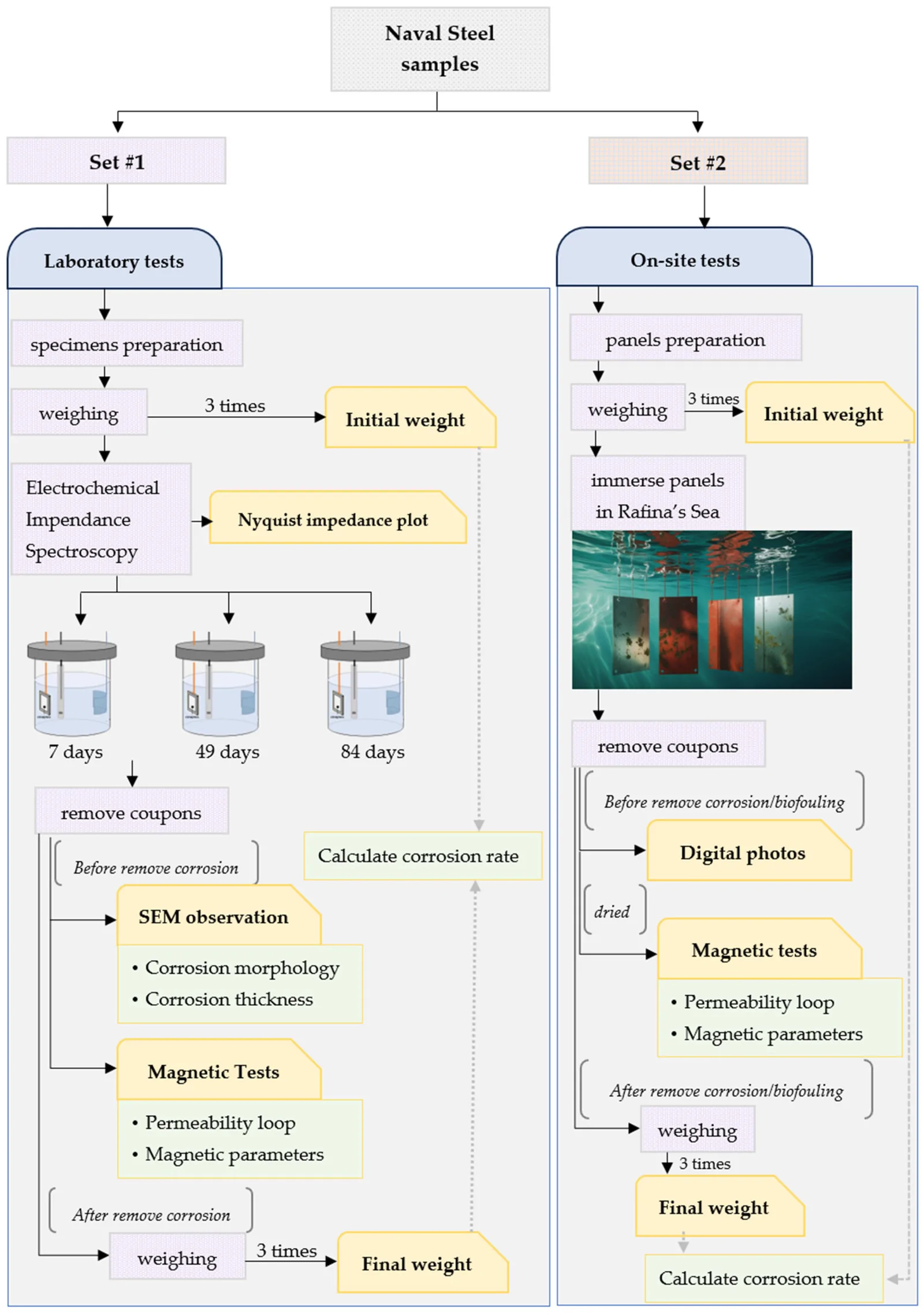
Diagram illustrating experimental procedure protocol.

Two sets of naval steel samples were prepared: one set for corrosion assessments in controlled laboratory artificial seawater (ASW) conditions and another set for evaluating corrosion resistance in situ under natural seawater conditions (Figure 2).

Figure 2 systematically presents the experimental protocol for evaluating corrosion in naval steel samples through a bifurcated approach: controlled laboratory tests (Set #1) and on-site exposure (Set #2). For Set #1, laboratory specimens undergo precise weighing (triplicate measurements) to establish initial mass, followed by electrochemical impedance spectroscopy for Nyquist plot acquisition. Samples are immersed in corrosive media for defined intervals (7, 49, and 84 days). At each endpoint, test coupons are extracted for sequential analyses including scanning electron microscopy (SEM) observation—yielding corrosion morphology and layer thickness—and magnetic testing for determining permeability loop and related magnetic parameters. Final weights are recorded (triplicate) after corrosion removal, culminating in corrosion rate calculation.

Concurrently, Set #2 involves onsite specimen preparation, repeated weighing, and immersion in natural seawater (Rafina’s Sea). Post-exposure, panels are retrieved for digital photographic documentation and dried for subsequent magnetic testing. After removing corrosion and biofouling, final weights are measured (triplicate), enabling corrosion rate computation. This integrated protocol allows comprehensive comparison between controlled and in situ corrosion degradation, leveraging advanced imaging, magnetic, and electrochemical methodologies to elucidate both quantitative and mechanistic aspects of steel corrosion behavior.

#### 2.2.1. Electrochemical Corrosion Tests

Prior to electrochemical testing, the steel samples were subjected to a systematic preparation protocol to ensure surface uniformity and the reproducibility of results. Specimens were mechanically abraded using silicon carbide papers of progressively finer grit, typically ranging from 320 to 1200, to achieve a smooth, oxide-free surface. Following mechanical polishing, the samples were thoroughly degreased in acetone using ultrasonic agitation to remove organic contaminants and particulate debris. Subsequently, the specimens were rinsed with deionized water and gently dried in stream ambient air. To confine the exposed area during testing, non-conductive lacquer was applied to all surfaces except the working face, which was precisely defined to a known area. This meticulous preparation minimized extraneous electrochemical activity and ensured that acquired impedance data accurately represented the intrinsic corrosion behavior of the steel in the test environment.

Electrochemical static immersion tests were conducted in ASW. The chemical composition of ASW was determined according to ASTM D1141 specifications to closely simulate average ocean water [23]. Table 4 prescribes the standard’s major constituents per liter of ASW solution.

After these salts were dissolved in distilled water and the solution was made up to 1 L total volume, the pH was adjusted to 8.2, using sodium hydroxide as necessary. Thus, the ASW prepared for ASTM D1141 standard contains all the principal ionic components of natural seawater in their typical proportions, ensuring relevance for corrosion applications.

According to ASTM G31-72 [24], the total volume of ASW required for a corrosion immersion test is determined by the ratio of solution volume to total exposed specimen surface area. The standard specifies using a minimum of 0.2 to 0.4 L of solution per square centimeter (cm^2^) of exposed metal surface. This ensures that the accumulation of corrosion products and dissolution of metal ions do not significantly alter the chemistry of the test solution during the experiment. For 9 cm^2^ total exposed area and using 0.3 L/cm^2^, the required solution volume was 9 cm^2^ × 0.3 L/cm^2^ = 2.7 L of ASW. Consequently, three 1 L solutions were formulated in accordance with ASTM D1141 requirements. Subsequently, only 2.7 L of ASW were employed, as specified in ASTM G31-72.

**Figure 3:**
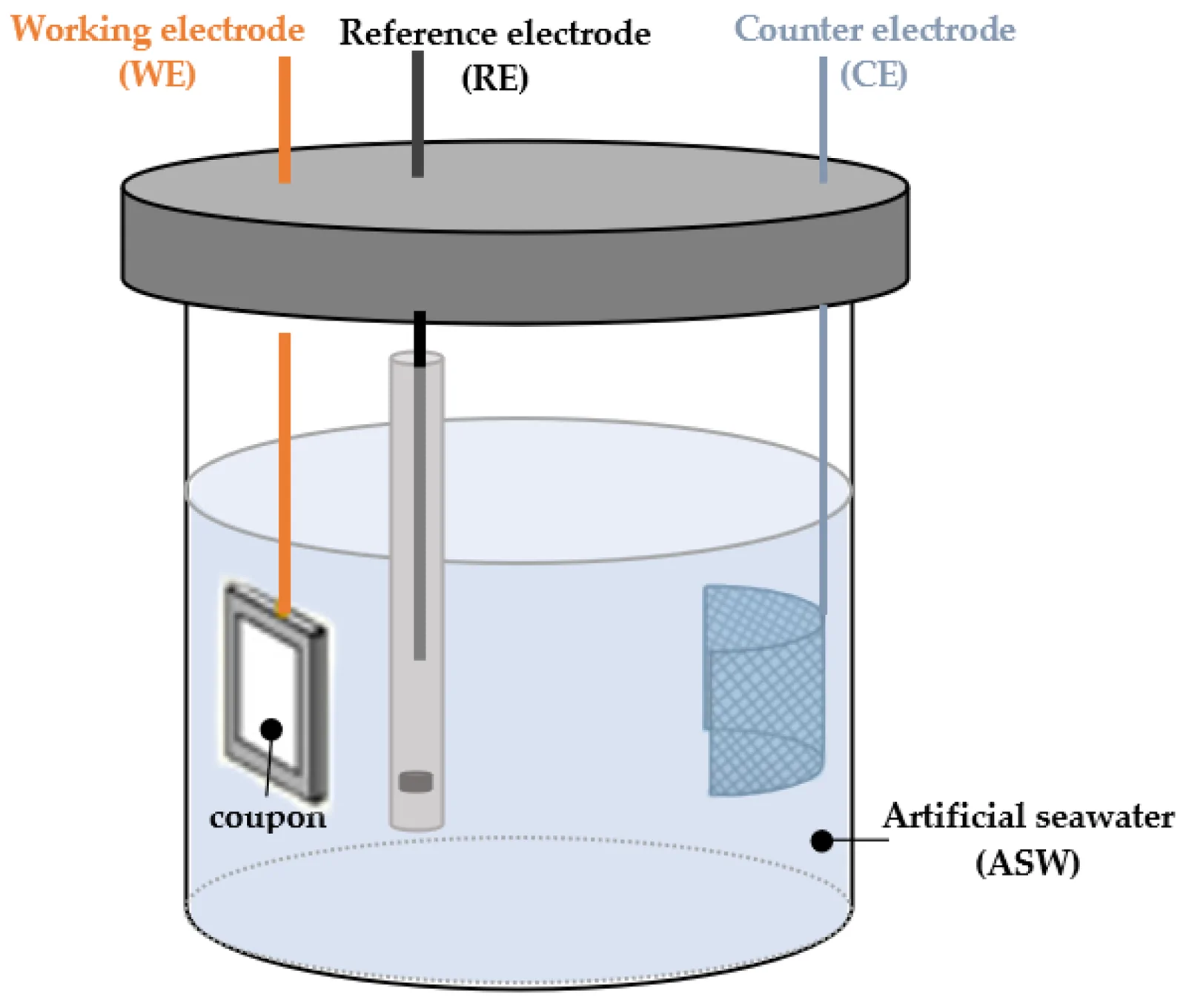
Apparatus used in laboratory electrochemical corrosion tests.

Three containers were utilized, each holding identical quantities and compositions of ASW [2]. A coupon was inserted into each container and kept there for specified durations: 7, 49, and 84 days. Electrochemical impedance spectroscopy (EIS) was carried out using a PGSTAT-302N potentiostat (Metrohm A.G., Herisau, Switzerland) and a three-electrode electrochemical cell, with a graphite rod serving as the reference electrode (RE) and silver–silver chloride (SCE)/(Ag/AgCl) acting as the counter electrode (CE). The steel specimens, whether uncorroded (0 days of immersion) or corroded (7, 49, and 84 days), served as the working electrode (WE) (Figure 3). Electrochemical impedance spectroscopy (EIS) experiments were conducted by applying a sinusoidal voltage of ±10 mV and achieving impedance responses in a frequency range of 10^5^ Hz to 0.1 Hz, with reference to the open circuit potential (OCP). All measurements were conducted at room temperature. Electrochemical data from EIS measurements were analyzed using NOVA 2.1.3 software (Metrohm A.G., Herisau, Switzerland).

Prior to the corrosion test, each of the three specimens was weighed three times and the average of these three weights was recorded as the pre-corrosion weight of the specimen. The specimens were placed into the seawater container. The corrosive solution in each container must be refreshed every five days to maintain the stability of its composition throughout the test. Specimens were collected following their stay at ASW, and the corrosion morphology of each specimen was examined prior to rust removal. Subsequent to rust removal, each specimen was weighed thrice, with the mean of the three weights used as the weight of the specimen post-corrosion (Figure 2).

Weight loss is the primary quantitative evaluation tool for analyzing the corrosion of naval steel [25]. The principle is founded on the alteration (reduction) in the weight of the material specimen before and after corrosion, signifying the loss of material mass [26]. The formula for computation is as follows:

(1) $$WL=\frac{w_{i}-w_{f}}{w_{i}}$$

where WL represents the weight loss ratio of the specimen, w_i_ denotes the weight prior to corrosion (g), and w_f_ indicates the weight subsequent to corrosion (g).

The evaluation of material corrosion rate often utilizes the weight loss method and the thickness reduction approach [27]. The weight loss method quantifies the weight reduction per unit surface area of the material over a designated time interval. This is determined with the following formula:

(2) $$CR=\frac{w_{i}-w_{f}}{A\cdot t}$$

where CR represents the corrosion rate of the specimen (g/(mm^2^·day)); A denotes the surface area of the specimen experiencing corrosion (mm^2^); and t indicates the time of the corrosion process (day).

Prior to the removal of corrosion products, a vertical cross-sectional study of the corroded samples was conducted using scanning electron microscopy (SEM, JEOL JSM-6490LV, Tokyo, Japan) to ascertain the thickness of the corrosion layer. Following the elimination of corrosion, the surface morphology of the naval steel was analyzed.

#### 2.2.2. Magnetic Measurements

**Figure 4:**
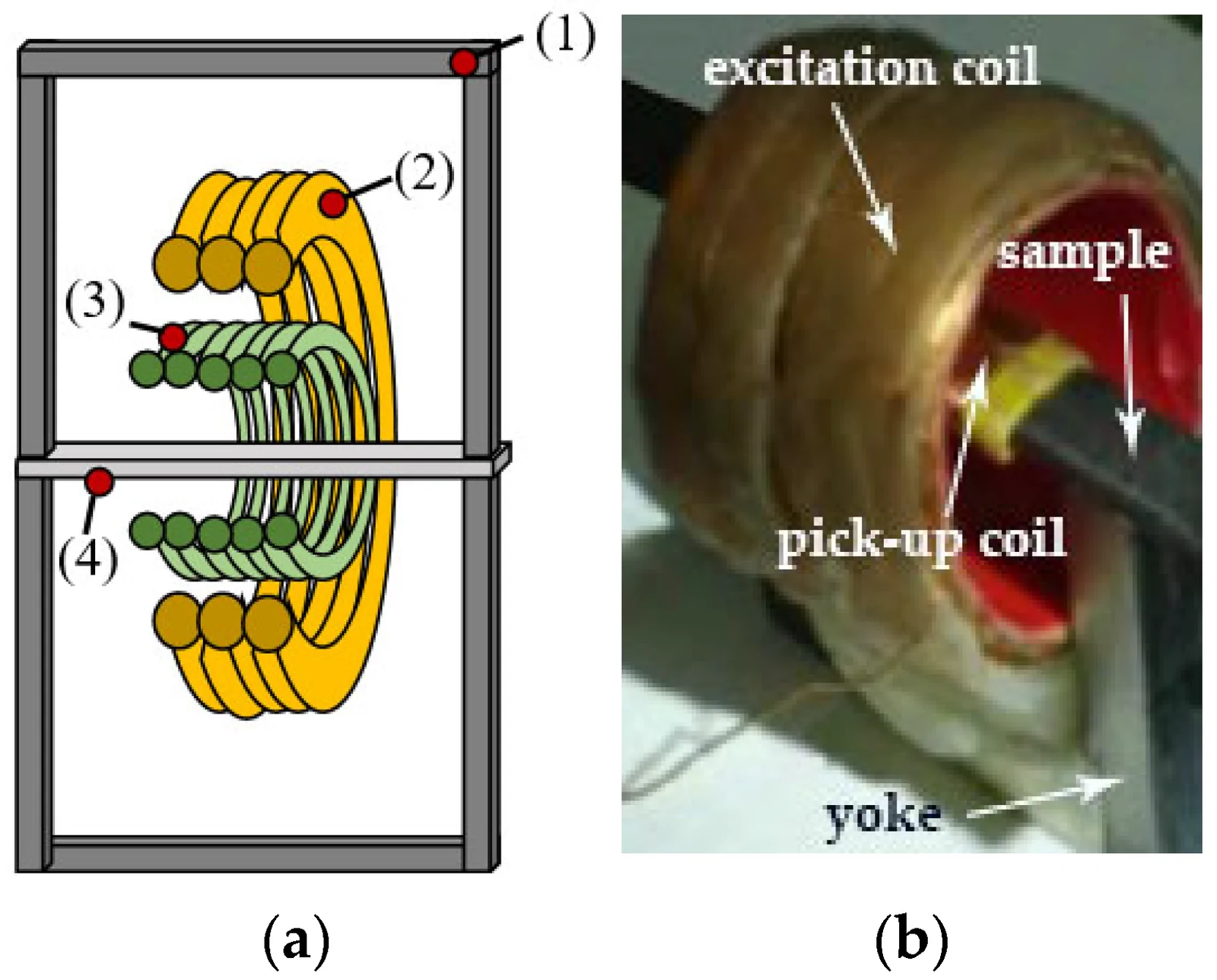
(**a**) Vertical sectional view of magnetic measurement setup using magnetic hysteresis magnetometer. Four components are (1) electromagnets, (2) 0.5 mm diameter copper wire excitation coil, (3) 0.2 mm diameter copper wire pick-up coil, and (4) ferromagnetic material that was put to test; (**b**) digital photo of coils.

Before the corrosion products layer was removed, the naval steel samples were assessed using the non-destructive magnetic permeability method. The magnetic measurements of the uncorroded and corroded samples were performed using an internal laboratory AC hysteresiograph (Figure 4).

**Figure 5:**
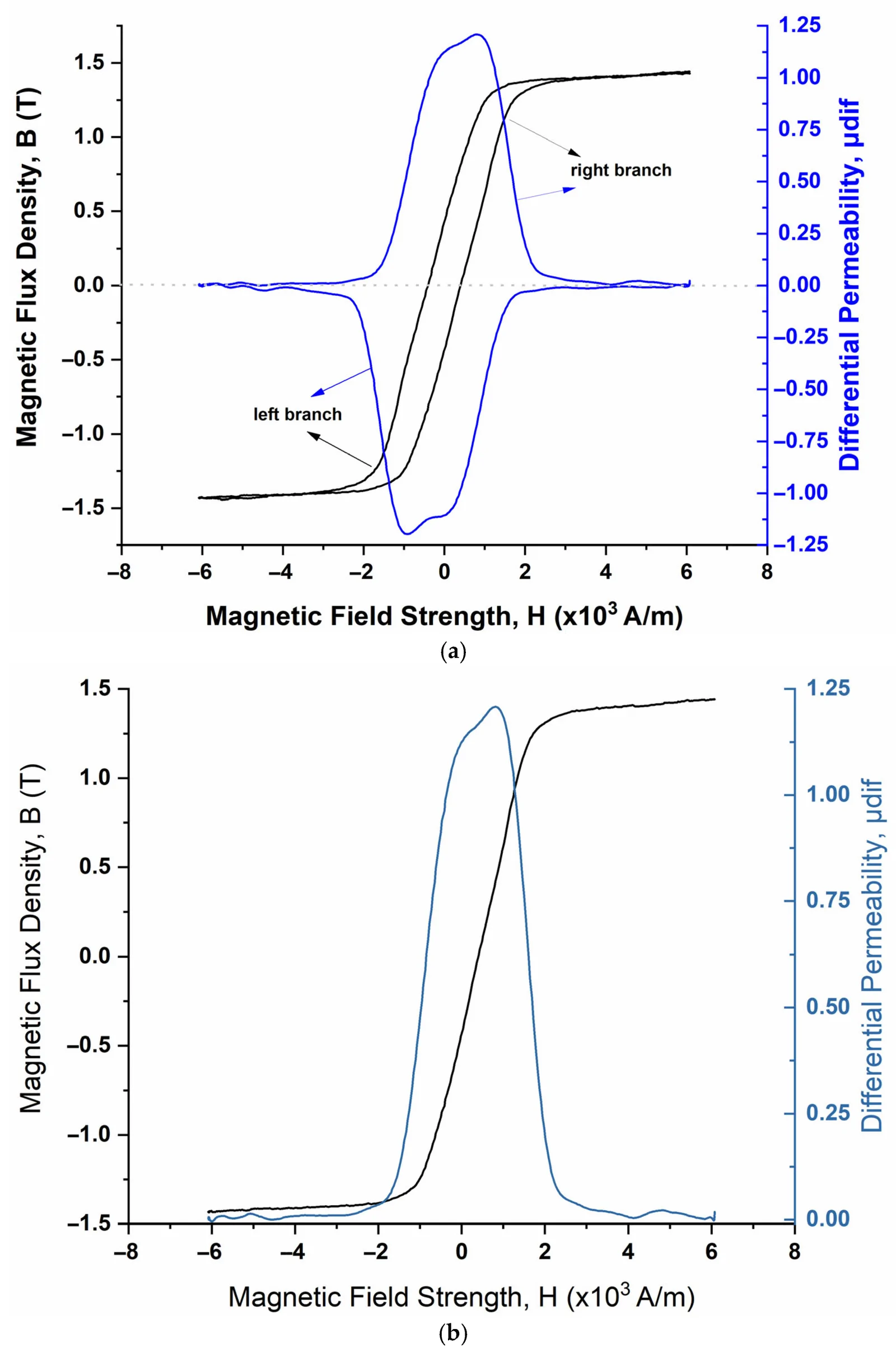

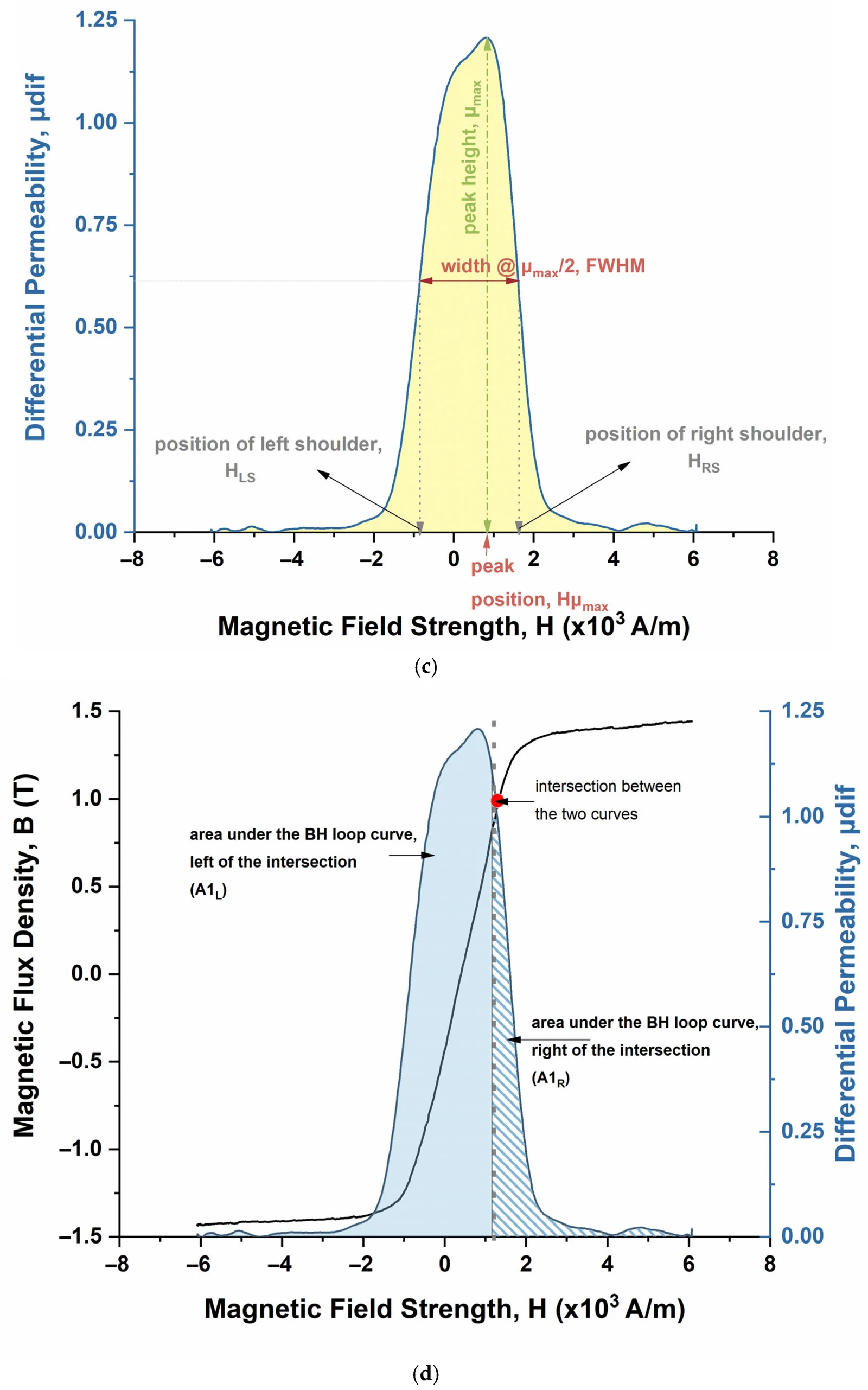
(**a**) µ_diff_-H curve and BH loop of isothermally annealed electrical steel sample; (**b**) right branch of BH loop and positive branch of µ_diff_-H curve; (**c**) diagram showing how to determine peak position and height, the peak width and the positions of the left and right shoulders at half peak height, and the total area under the μ_diff_-H curve; (**d**) area under the BH loop curve, left and right of the intersection.

The sample (denoted as (4) in Figure 4a) is situated within an excitation coil (identified as (2) in Figure 4a), while a sensing coil (marked as (3) in Figure 4a) encircles it (Figure 4b). A double yoke (denoted as (1) in Figure 4a) is employed to complete the magnetic circuit and mitigate demagnetizing effects [28]. The excitation coil received a low-frequency sinewave signal (0.1 Hz) to ensure uniform magnetic flux penetration through the examined thick ferromagnetic steels. The voltage pulse produced at the terminals of a sensing coil manifests as a sinusoidal waveform, exhibiting the same frequency as the original signal, altered by the magnetization response of the sample volume within the sensing coil. The demodulated output pulse is directly proportional to the disparity in permeability of the material [28,29]. The output pulse was integrated into the excitation field, yielding the hysteresis loop (black loop in Figure 5). A self-developed software was utilized to document the magnetic parameters, specifically the permeability profile (μ_diff_-H curve) and the applied field corresponding to the maximum output voltage, referred to as the coercivity field [28].

Figure 5a illustrates the μ_diff_-H curve obtained from a naval steel sample, together with the corresponding hysteresis loop. The diagram illustrates that the differential magnetic permeability reaches its peak near the coercive field within the hysteresis loop. Owing to the symmetrical properties of the hysteresis loop and the μ_diff_-H curve, the focus was mostly placed on the right side of the hysteresis loop, aligning with the positive branch of the μ_diff_-H (Figure 5b). The μ_diff_-H curves were assessed within the magnetic field strength range of (−6–+6) × 10^3^ A/m. This evaluation entailed computing various summary metrics, such as the peak position and height (Figure 5c), the peak width, and the positions of the left and right shoulders at half peak height (Figure 5c), the total area beneath the positive branch of the μ_diff_-H curve (Figure 5c), and the area under the curve to the left and right of the intersection point of the two plots (Figure 5d).

The intersection of the two curves, show in BH and μ_diff_-H plots (Figure 5b), delineates two regions, one to the left and one to the right of the crossing point (Figure 5d). The area below the graph (Figure 5d), on either side of the junction point, is linked to the irreversible process of magnetization [30]. To the right of the intersection of the two curves, the magnetic field values are elevated and can orient the magnetic regions parallel to the external magnetic field. Moreover, in this area, the nucleation and proliferation of reverse domains at grain boundaries has been confirmed [31]. Consequently, the area of the BH curve to the right of the junction (A1_R_) corresponds to the energy necessary to align the magnetic domains with the external field.

Similarly, to the left of the intersection of the two curves, the magnetization is defined by irreversible domain wall motion, including either 180° or 90° domain wall displacement [30]. The magnetic permeability intensity around the coercivity point is notably high and is defined by 180° domain wall motion [32]. The decline in magnetic field values correlates with the diminished amplitude of μmin subsequent to the original peak, which is associated with the displacement of 90° domain walls. The area of the BH curve to the left of the intersection (A1_L_) corresponds to the energy required for irreversible domain wall motion, whether through 180° or 90° domain wall displacement, primarily due to the pinning effect of microscopic defects in materials, such as dislocations, voids, and grain boundaries, which are generally linked to material processing technology [32].

#### 2.2.3. In Situ Static Immersion Tests

**Figure 6:**
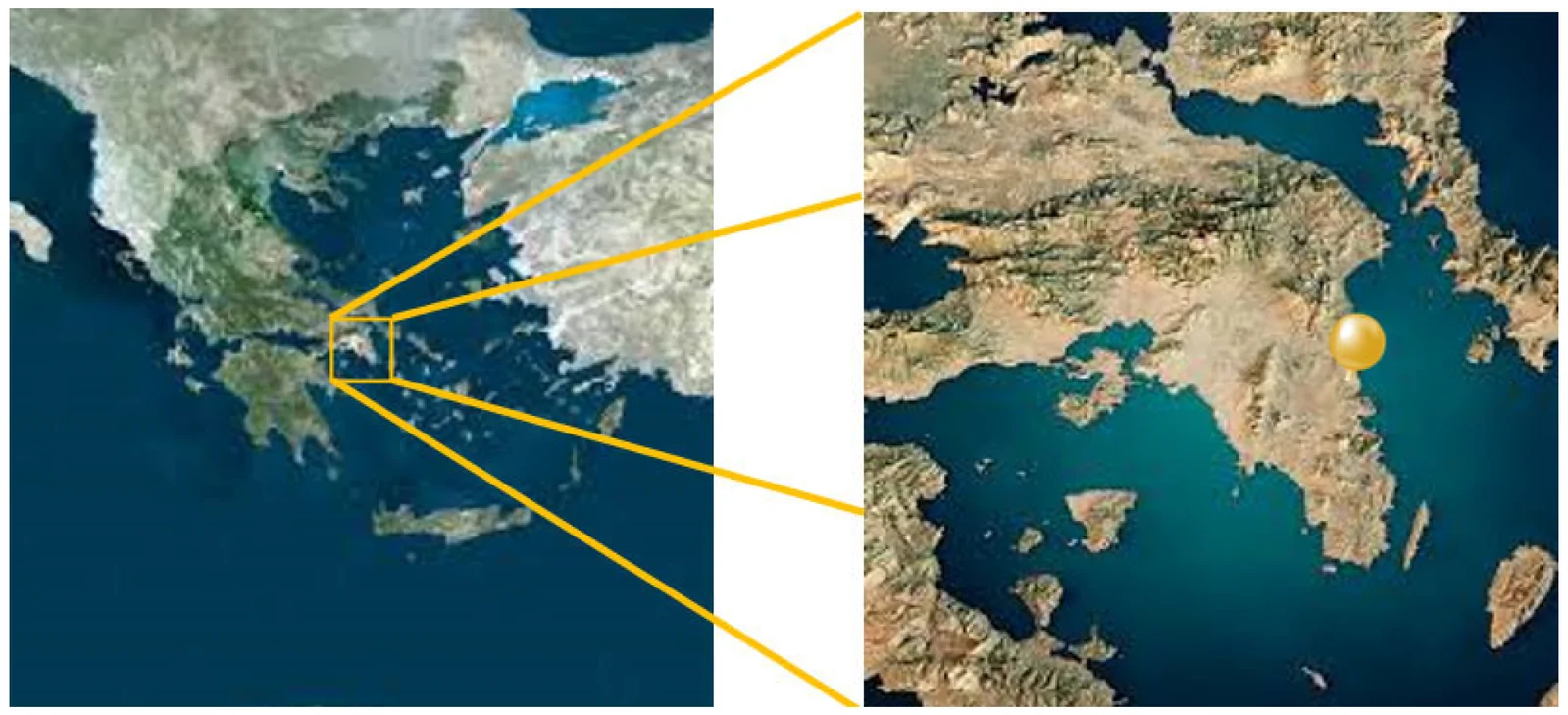
Satellite map of Greece showing the location selected for in situ immersion tests.

At the conclusion of February 2025, three naval steel panels were immersed at a depth of approximately 1 m in the natural sea water for differing durations of 7, 49, and 84 days. The chosen site was adjacent to the coast of Rafina (Figure 6).

Upon concluding their immersion in natural seawater, the samples were meticulously extracted. The macroscopic visual examination of the samples, obtained by digital image downloads, revealed the extent of surface biofouling.

Subsequently, the surface of the collected naval steel panels was purged of its current fouling level. Following the drying process in the laboratory, magnetic measurements were conducted, yielding magnetic permeability loops and parameters, as illustrated in Figure 5c,d. The magnetic characteristics were associated with the concentrations and corrosion rates of the samples submerged in natural seawater.

## 3. Results

### 3.1. Electrochemical Impedance Spectroscopy (EIS) Tests

To generate varying degrees of corrosion in naval steel and to analyze the corrosion behavior of the samples investigated, electrochemical impedance spectroscopy (EIS) measurements were conducted.

**Figure 7:**
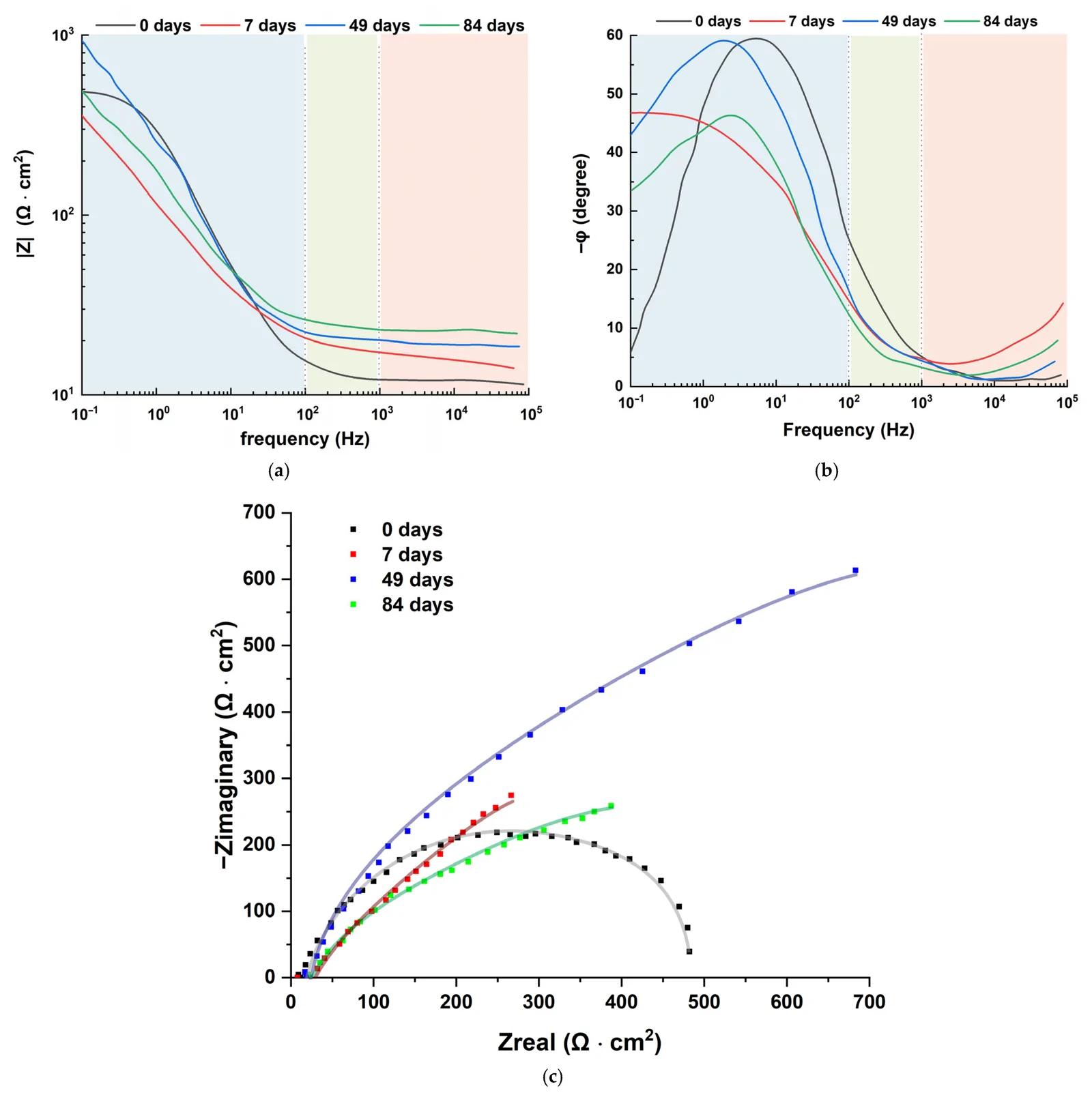
EIS diagrams of the naval steel specimen submerged in ASW for varying durations. (**a**) Bode diagram of impendence as a function of frequency; (**b**) Bode diagram of phase angle as a function of frequency and (**c**) Nyquist diagrams (solid lines present the fitting curves). In Figure 7a,b, the blue region denotes low frequency areas, the light green regions signify medium frequency areas, and the orange region indicates high frequency areas.

Data on the real and imaginary components of impedance were obtained for each voltage frequency during the EIS studies [33]. The EIS diagrams, comprising the Bode (Figure 7a,b) and Nyquist (Figure 7c) diagrams, were derived from these measurements [34]. The Bode diagrams consist of an amplitude (gain)–frequency plot (Figure 7a) and a phase–frequency plot (Figure 7b) [35]. The Nyquist diagrams (Figure 7c) depict the gain and phase of the frequency response (Bode diagrams) in the complex plane using polar coordinates. The angle of a point relative to the origin signifies the phase, whereas the distance from the origin denotes the gain [3].

Analyzing the EIS diagrams (Figure 7), there are specific zones that define the electrochemical behavior of the samples of concern (Table 5).

The Bode plot indicates that the peak impedance value at low frequencies is observed after 49 days of steel immersion in ASW (Figure 7a). Upon examining the impedance values throughout all immersion durations, the subsequent sequence is derived: |Ζ|_49_ > |Ζ|_84_ > |Ζ|_7_ > |Ζ|_0_. Since a high impedance value signifies a reduced corrosion rate and enhanced corrosion resistance, it is evident that the steel sample subjected to short immersion durations (0–7 days) demonstrates elevated corrosion rates. The corrosion rate drastically drops after 49 days and then continues to decline. The phase angle Bode plots (Figure 7b) indicate that on the 49th day of residency in ASW, the phase angle approaches 90°, signifying enhanced inhibitive performance.

The naval steel specimens corroded in ASW exhibit incomplete semicircles in the Nyquist plots (Figure 7c). The rays from the spatial arcs adhere to the subsequent sequence: R_49_ > R_84_ > R_7_ > R_0_.

**Figure 8:**
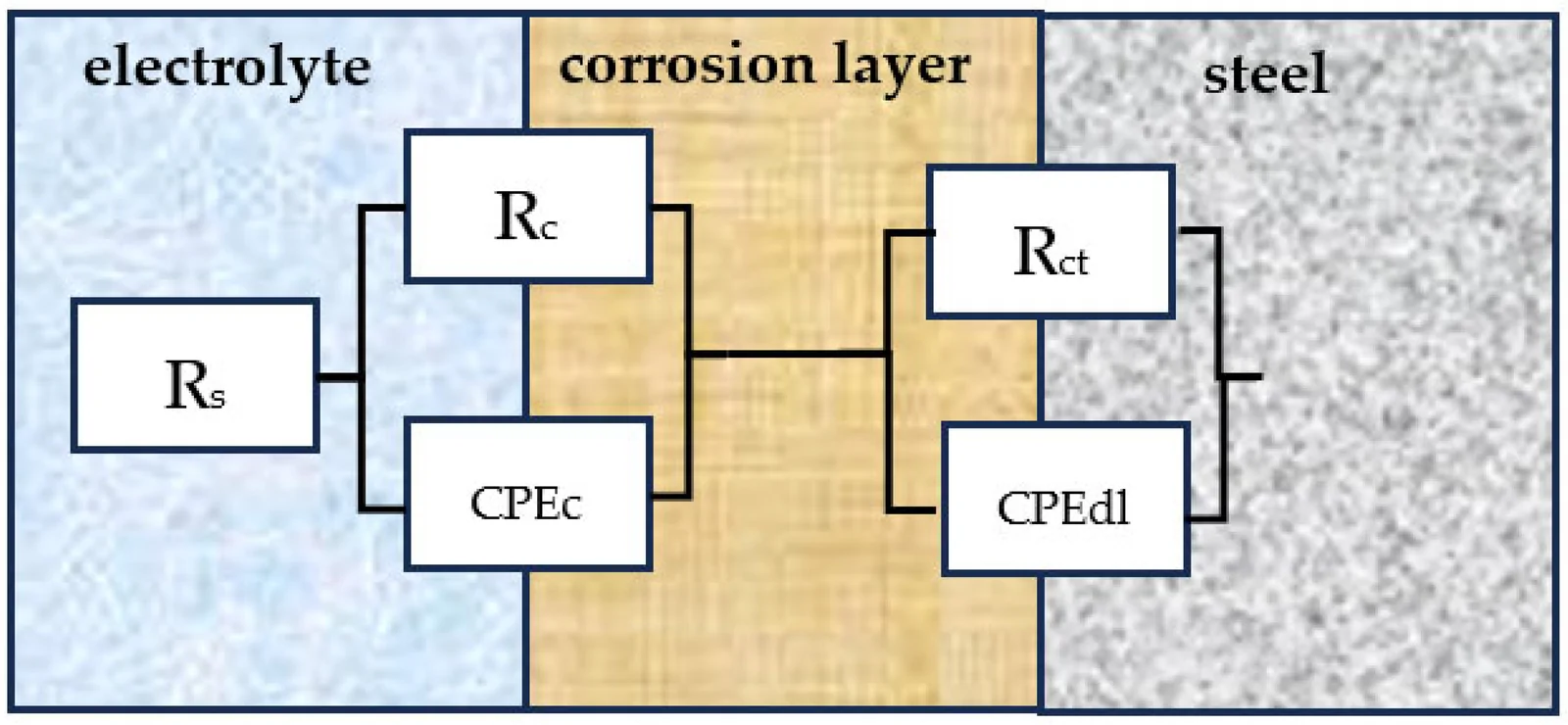
Electrical equivalent circuit of electrochemically corroded steels.

The steel sample’s plots can be represented by the electrical equivalent circuit depicted in Figure 8. It comprises the electrolyte resistance (*R*_s_), the charge transfer resistance (R_ct_), and the constant-phase element (CPE_dl_) that represents the double layer, the corrosion layer capacitance (CPE_c_), and the corrosion layer resistance (R_c_).

The selection of the electrical equivalent circuit depicted in Figure 8 is essential for accurately modeling the experimentally observed EIS data presented in Figure 7c. In complex systems such as corroding naval steels immersed in ASW, the impedance response is influenced by multiple interfacial processes, including charge transfer at the steel interface, mass transport through the corrosion layer, and dielectric properties of the evolving corrosion products. The equivalent circuit in Figure 8 incorporates separate elements for the solution resistance (R_s_), resistance and constant-phase elements of the corrosion layer (R_c_ and CPE_c_), and resistance and constant-phase elements of the double layer at the steel interface (R_ct_ and CPE_dl_). This multi-component approach allows for the deconvolution of processes occurring within the corrosion product layer and at the bare metal/electrolyte interface, which manifest as multiple time constants in the EIS spectra. The chosen configuration reflects established models for advanced EIS analysis in corrosion science, which require recognition of heterogeneous, multilayered surface structures to achieve physically meaningful fits [36,37].

Simpler equivalent circuits, such as those consisting only of solution resistance, a single-charge transfer resistance, and a single-capacitance or constant-phase element, might suffice for pristine or uniformly corroding systems. However, the EIS plots in Figure 7c display behaviors—particularly at longer immersion times—indicative of at least two overlapping semicircular arcs or depressed arcs in the Nyquist representation. This response suggests two (or more) relaxation processes consistent with a layered structure: an outer, porous or partially resistive corrosion film, and an inner, reactive interface where electron transfer occurs. The presence of a distinct R_C_-CPE branch for the corrosion layer in Figure 8 enables the model to capture the frequency-dependent impedance associated with the non-ideal, inhomogeneous nature of real corrosion films—features that are not adequately addressed by single-time-constant circuits [36,38].

The appropriateness of the chosen Figure 8 circuit is further validated by contemporary literature. For example, Zhang et al. demonstrated that multi-time-constant circuits—incorporating both resistance and constant-phase elements for separate physical layers—significantly improved the fit with experimental EIS data from corroded steel in chloride-containing environments. Their analysis shows that the additional R_C_–CPE parallel pairs account for distributed capacitance and resistance arising from diffusion, porosity, and heterogeneity in the oxide/oxyhydroxide corrosion products [39]. Similarly, Feliu highlights that such modeling strategies reliably extract mechanistically relevant corrosion parameters, such as film resistance and charge transfer resistance, from layered corroded electrode systems [40]. As such, the circuit in Figure 8 not only achieves a closer empirical fit but also conveys a more physically meaningful representation of the corrosion system’s electrochemical architecture, supporting both qualitative interpretation and quantitative extraction of corrosion parameters over exposure time.

The parameter values for the optimal fit of the experimental impedance plots are displayed in Table 6.

The comparison of *R*_ct_ values for samples subjected to varying corrosion durations in ASW, as presented in Table 5, indicates that the lowest *R*_ct_ occurs on day 0 due to the absence of a rust layer on the surface of the immersed sample. As immersion duration grows, *R*_ct_ rises and attains its peak value on day 49 of immersion. A further reduction in the *R*_ct_. value is seen. Comparable behavior is noted for R_c_. Table 5 indicates that during the initial phases of immersion, the values of CPE_dl_ and CPE_c_ rise, followed by a subsequent decline. The diminishing values signify a decline in the presence of hostile ions on the steel surface.

The values of n_c_ and n_dl_ in Table 5 represent the exponents of the constant-phase element (CPE) in the equivalent electrical circuit used to fit the electrochemical impedance spectroscopy (EIS) data for DH36 steel immersed in ASW. These exponents are critical because they quantitatively describe the deviation of the interfacial response from ideal capacitive behavior at both the corrosion layer (n_c_) and the electrical double layer (n_dl_). An exponent value of 1 signifies a behavior close to that of an ideal capacitor—indicative of a smooth, homogeneous, and defect-free interface. However, values less than 1 reflect non-ideal capacitance often attributed to surface roughness, heterogeneity, porosity, and localized processes at the steel/electrolyte interface [41,42,43].

In this study, both n_c_ and n_dl_ exhibit dynamic variations with immersion time: n_c_ starts lower (0.60) at 7 days, rises to 0.75 at 49 days, and slightly drops to 0.66 by 84 days, while n_dl_ increases gradually from 0.87 to 0.95 over the same period. The initial low values indicate that the early corrosion stages promote a highly heterogeneous and porous surface, likely due to rapidly nucleating and loosely bound corrosion products. As exposure advances, the corrosion layer densifies, and the CPE exponents increase, approaching more ideal, capacitive behavior—this is characteristic of a more homogenous, compact, and protective corrosion product layer. At longer times (84 days), the minor decline or plateau in n_c_, alongside the continued increase in n_dl_, suggests the formation of microcracks, increased porosity, or localized defects in the thickened film, with the double layer itself becoming more uniform as the overall system stabilizes. This evolution in n_c_ and n_dl_ thus directly mirrors the physical and chemical changes in the steel/corrosion layer interfaces, linking EIS-derived parameters to microstructural transformations and corrosion protection mechanisms [44].

The chemical transformation of the steel surface is thus dynamic, driven by redox reactions and evolving transport limitations within the corrosion film. Early stages feature the rapid formation and transformation among less stable iron oxyhydroxides, governed by high pH and oxygen supply at the steel surface. Over time, the transition to more crystalline and stable rust phases reduces the mobility of aggressive ions but does not preclude under-film corrosion, particularly as occluded cell conditions develop. This temporal evolution in composition and morphology reflects changing electrochemical gradients and can be monitored by a combination of analytical techniques such as SEM and XRD.

**Figure 9:**
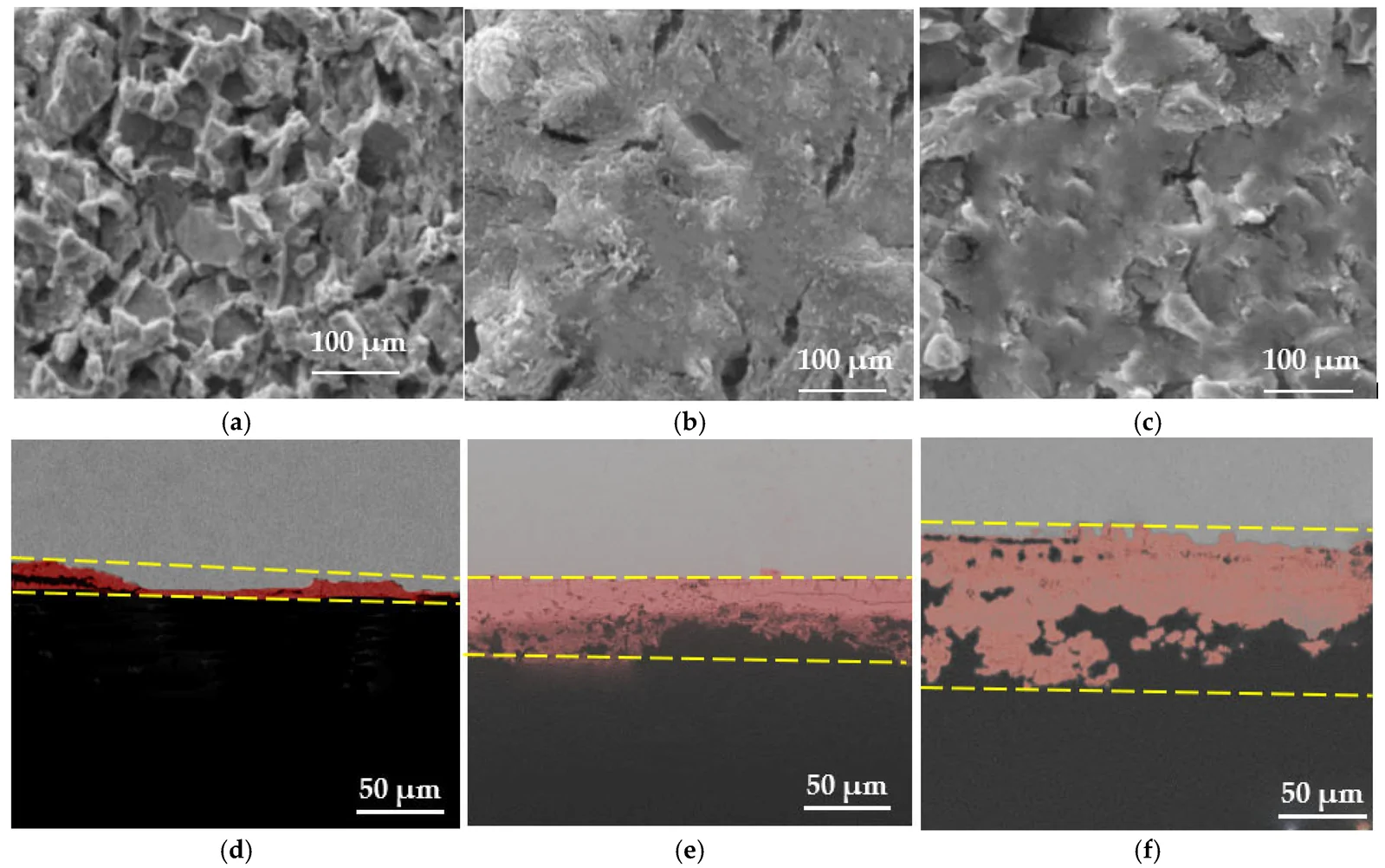
SEM micrographs depicting (**a**–**c**) the surface and (**d**–**f**) corresponding cross-sections following electrochemical impedance spectroscopy measurements. The corrosion layer is depicted in red, and the region between the yellow dotted lines delineates the thickness of the corrosion products on the surface of the naval steel.

Upon examining the surface and vertical cross-section of the corroded specimens prior to the removal of the corrosion layer, it is evident that on the seventh day of immersion, loose clusters of granular morphology and the heterogeneous dispersion of corrosion products are present on the steel surface (Figure 9a). As residence time increases, the corrosion products aggregate and develop in a network distribution (Figure 9b). Following 84 days of habitation in ASW, the corrosion layer deteriorates, resulting in corrosion products forming irregular network blocks (Figure 9c).

The coating layer’s thickness increases with the duration of exposure to ASW (Figure 9d–f). A thin, discontinuous layer is initially produced on the steel substrate (Figure 9d). The layer of corrosion products increases in thickness and density, conforming to the topographical contours of the steel interface (Figure 9e). With extended residence times, the corrosion products develop a relatively thick layer characterized by cracking and significant porosity (Figure 9f).

Corrosion layers formed in artificial seawater—especially over extended durations—may appear thicker but more adherent, while those developed in variable-salinity settings [45] often show more rapid spallation and microcrack formation, visible in SEM as fractured or flaking scales. As P. Cheng and X. Huang discussed [45], increased salinity accelerates both corrosion rate and the formation of more porous, layered corrosion products, often characterized by uneven surface features, pitting, and higher product delamination. These microstructural signatures reflect the pronounced interplay between chloride ion concentration and the breakdown of protective films on naval (DH36) steel surfaces.

In contrast, Figure 9 of the current manuscript, depending on its experimental protocol and immersion environment, likely displays corrosion morphologies induced under controlled laboratory conditions. The differences in corrosion product compactness, pit density, and layer adherence compared to the SEM images of P. Cheng and X. Huang [45] can be attributed to variation in factors such as exposure time, temperature, or oxygen access. Methodological divergences further account for observable differences. Figure 9 aggregate findings across multiple samples or time points, potentially highlighting average corrosion progression, while the SEM images of P. Cheng and X. Huang [45] focus on detailed microscale features at specific salinity levels. As a result, the P. Cheng and X. Huang SEM imagery [45] can be expected to capture sharper evidence of localized attack—such as discrete pitting nuclei or salt crystal inclusions—whereas Figure 9 could offer a broader depiction of uniform versus localized corrosion morphologies. Together, these differences underscore the influence of environmental salinity, immersion protocol, and analytical scale on corrosion manifestation and its subsequent documentation through SEM imaging.

**Figure 10:**
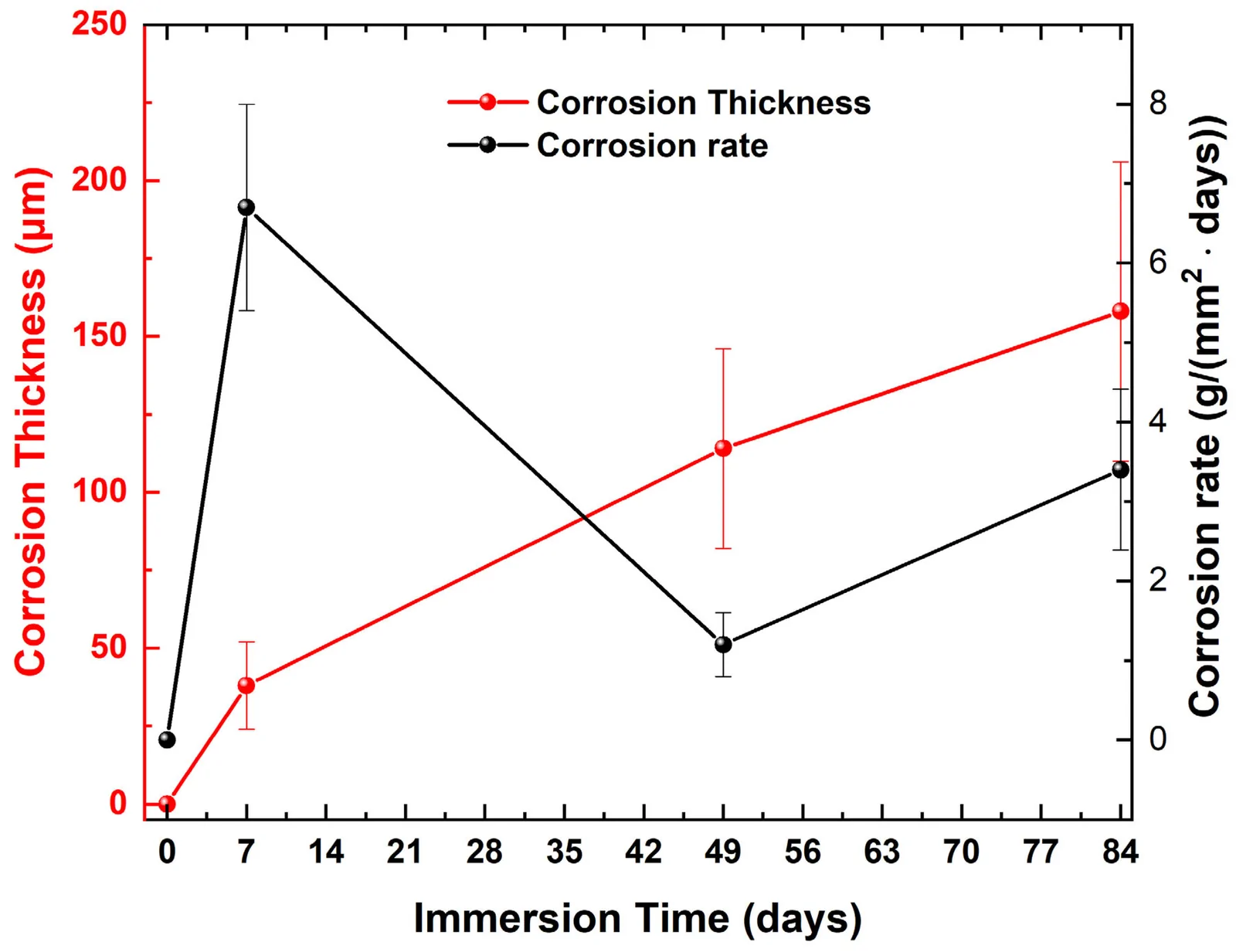
Diagram illustrating difference in corrosion layer thickness and surface corrosion rate of naval steel.

After extraction from the containers, each of the three corroded samples (7, 49, and 84 days) was dried and subsequently sectioned with a microtome. The estimated corrosion thickness was the average of seven measurements obtained from cross-sectional SEM images. Figure 10 illustrates the fluctuation in the thickness of corrosion products and the corrosion rate, as ascertained by the weight loss method (Equation (2)). Figure 10 clearly illustrates the evolution of both corrosion layer thickness and the surface corrosion rate of naval steel over an 84-day immersion period. Despite the progressive growth in the thickness of the oxide layer, the corrosion rate does not display a monotonic pattern. The results show an initial rapid increase in corrosion rate, peaking by day 7, followed by a pronounced decline by day 49. This is then succeeded by a secondary increase up to day 84, but with a lower peak than on day 7. In contrast, the corrosion layer thickness demonstrates a steady, near-linear increase over the entire testing duration, suggesting continual oxide deposition even as the instantaneous corrosion rate fluctuates.

At the outset (0 days), the surface of the naval steel exhibits no oxide layer; consequently, the oxygen present on the sample’s surface is adequate, allowing for the quick diffusion of iron ions from the steel surface. This process persists until a coating of corrosion products forms on the steel surface, which happens by the seventh day of immersion (Figure 9a,d). During these brief residence intervals, the corrosion rate is at its peak (Figure 10). By the 49th day of immersion, a dense and compact corrosion layer has developed on the steel surface (Figure 9b,e), which protects the steel by inhibiting the diffusion of iron ions (generated during the anodic corrosion reaction) into the seawater [2]. The corrosion rate diminishes (Figure 10), and the sample exhibits comparatively inadequate corrosion resistance on the 49th day of immersion. The results are corroborated by the elevated immersion values and diminished capacitance values in the electrical circuit modeling electrochemical corrosion (Figure 8).

As the immersion time extends to the 84th day, the corrosion products exhibit weak adhesion to the substrate (Figure 9c), and the resultant corrosion layer on the specimen’s surface displays significant cracking and porosity (Figure 9f), thereby diminishing the protective efficacy (barrier property) of the corrosion layer on the steel. The presence of capillary phenomena facilitates the transport of iron ions into seawater, hence increasing the corrosion rate.

This experimental pattern is consistent with the fundamental model of marine corrosion described by R. Melchers [46], who proposed distinguished kinetic regimes: an initiation phase with an abundance of reactants resulting in high corrosion rates, followed by a period dominated by oxygen diffusion through the corrosion products, which slowed the process, and ultimately, a potential reignition of corrosion due to the establishment of anaerobic or microbially influenced conditions within the mature rust layer. The sharp decrease observed in the corrosion rate after the initial surge can be attributed to the formation of a compact corrosion product film that impeded oxygen access to the steel surface. The later upturn in rate, concurrent with significant thickening, likely marked the onset of localized breakdowns, where evolving rust microstructure and possibly microbial activity created renewed pathways for aggressive attacks [46].

When these findings are compared with prolonged marine immersion studies, such as those by Vukelic, Goran et al., on AH36 steel [47], a similar non-linear trend is observed. Their research documented that mass loss and corrosion rates were most pronounced between the early to mid-immersion intervals (up to 6 months), after which the rate declined and tended toward stabilization in subsequent months. Vukelic and colleagues reported that exposure to natural seawater environments caused significant corrosion, especially during initial months, corresponding to periods of rapid pit nucleation and growth, before the consolidation and thickening of the surface layer shifted the process toward more stable long-term degradation rates [47,48]. Their measured average corrosion rates for AH36 steel ranged from approximately 0.10 to 0.15 mm/year post-stabilization, in line with predicted steady-state values for marine steels in immersion conditions.

Furthermore, the increasing corrosion thickness in the presented study aligns closely with Vukelic et al.’s documentation of progressive microstructural damage. Scanning electron microscopy of long-term exposed AH36 samples revealed the coarsening and merger of corrosion pits—a process mirrored by the steady thickening observed in the current figure—along with the formation of dense, layered corrosion products. This reinforces the notion that, over time, the balance between corrosion layer build-up and loss due to spallation or pit coalescence governs the net growth of corrosion product thickness, as highlighted in empirical marine degradation models [47,48].

Integrating Melchers’ multi-phase framework [46] provides the mechanistic underpinning for these observations: the initial period is reaction-controlled, driven by exposed clean metal and rapid ion exchange, yielding high corrosion rates and relatively thin corrosion products. As revealed in Figure 10, this is quickly succeeded by a diffusion-controlled regime, where oxygen must penetrate the increasingly impermeable oxide scale—a process that sharply limits the mass loss rate but does not halt it entirely, allowing continual, albeit slower, thickening. With further exposure, the maturing rust layer creates localized occluded environments, susceptible to anaerobic reactions or microbial colonization, which can reaccelerate dissolution locally and reshape pit morphology and corrosion front stability.

In summary, the correspondence between Figure 10 and both Melchers’ corrosion phases [46] and Vikelic et al.’s long-term exposure [47,48] results substantiates a universal progression in the corrosion of marine steels. The findings highlight the initial dominance of rapid corrosion, the protective but ultimately permeable nature of corrosion scales, and the long-term interplay of kinetic and microenvironmental factors that together dictate material degradation trajectories in marine systems. These insights are critical when using empirical data to predict service life, inform maintenance schedules, or design protective systems for naval steel infrastructure.

**Figure 11:**
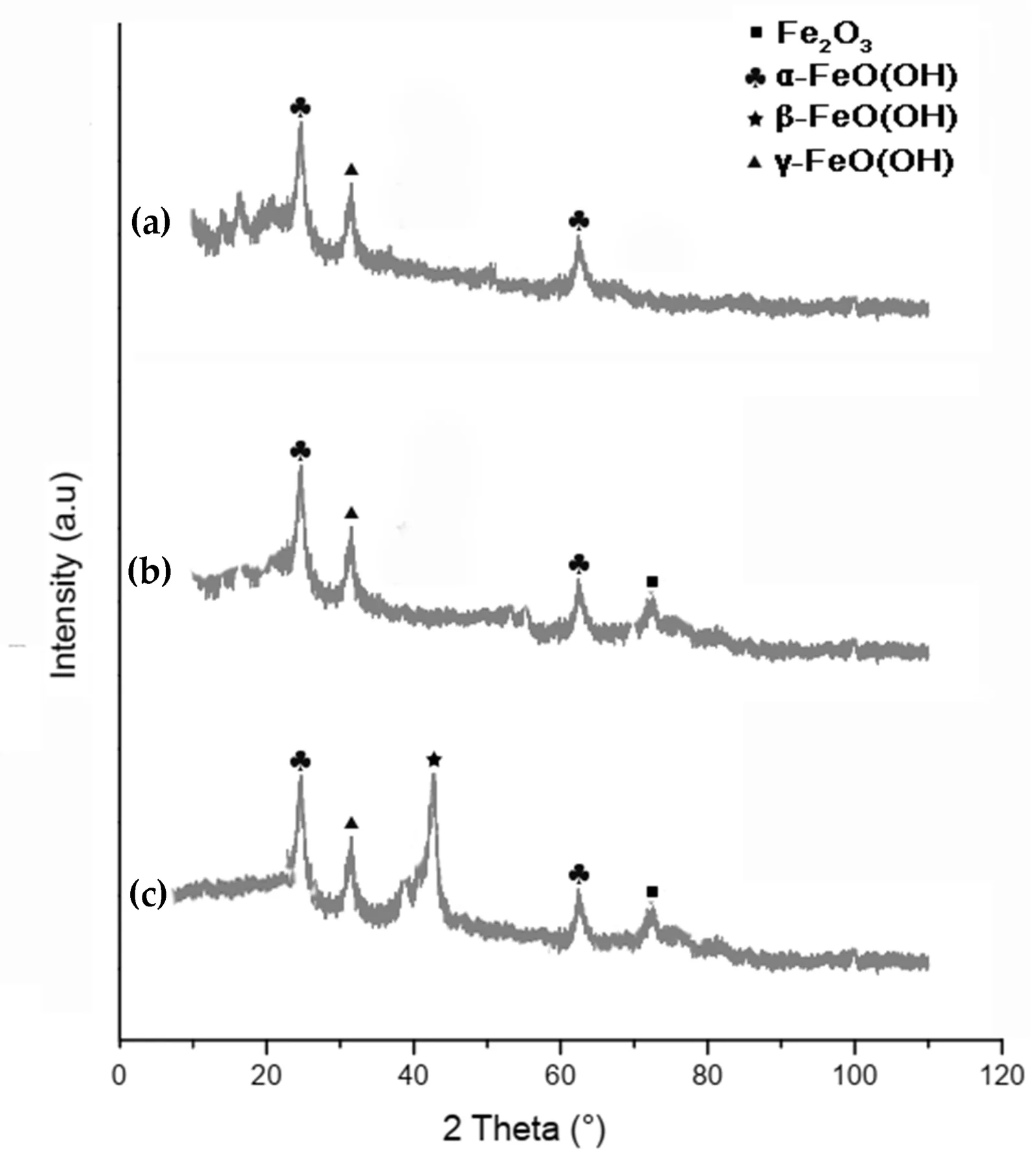
XRD pattern of t corroded naval steels immersed in ASW for (**a**) 7 days, (**b**) 49 days, and (**c**) 84 days.

XRD results (Figure 11) show that within the first week (7 days), initial corrosion is characterized by the formation of thin, poorly adherent films, composed mainly of lepidocrocite (γ-FeOOH) and goethite (α-FeOOH) (Figure 11a). These phases nucleate rapidly under oxygen-rich and chloride-laden conditions provided by artificial seawater, resulting in a granular, often porous surface [49]. At this stage, the corrosion film offers minimal protection, leaving the steel substrate vulnerable to aggressive, localized attack, as is common during the induction period of marine corrosion [50,51].

By 49 days of immersion, the corrosion products display increased complexity (Figure 11b) and layering. The film becomes noticeably thicker and more tightly adherent, with the continued presence of lepidocrocite and a growing proportion of goethite in the outer regions [52]. The inner parts of the rust layer, especially in microenvironments with lower oxygen availability, demonstrate the substantial formation of maghemite (γ-Fe_2_O_3_), denoting possible partial oxygen depletion beneath the compacting oxide [53]. The denser and more crystalline nature of these products impedes ion movement to the metal surface, explaining the observed reduction in the general corrosion rate after the initial burst [53].

When exposure is extended to 84 days, further stratification and phase evolution become apparent. Advanced analyses reveal the emergence of akaganeite (β-FeOOH), a chloride-containing iron oxyhydroxide [54]. Akaganeite’s acicular morphology is favored under conditions of persistent chloride ingress, which is typical for artificial seawater environments. The continuous thickening of the rust layer at this stage generally improves the barrier characteristic of the corrosion products, but internal porosity and microcracks offer localized sites for renewed attack, particularly under diffusion-controlled or partially anaerobic microenvironments—an effect described in Melchers’ multi-phase corrosion theory well [46].

It is important to note that the microscale heterogeneity of corrosion product layers increases with time. After 84 days, inner layers may contain mixtures of maghemite and goethite, while outer regions may contain lepidocrocite, akaganeite, and hematite, (Figure 11c). These spatial differences across the rust layer influence not only the mechanical integrity but also the electrochemical reactivity of the steel interface, with implications for both localized attack (e.g., pitting) and longer-term durability.

In conclusion, the corrosion products formed on naval steel immersed in artificial seawater progress through a well-established sequence: from initial lepidocrocite and goethite at early stages (7 days), through the emergence of compact goethite and maghemite layers (49 days), to the development of highly stratified films containing significant akaganeite and hematite (84 days). These transformations reflect the dynamic interplay of environmental factors, continuous phase transitions, and evolving microenvironments at the metal/corrosion product interface. Such observations are consistent with experimental findings in the literature and underscore the importance of time-resolved analysis of marine steel corrosion phenomena.

**Figure 12:**
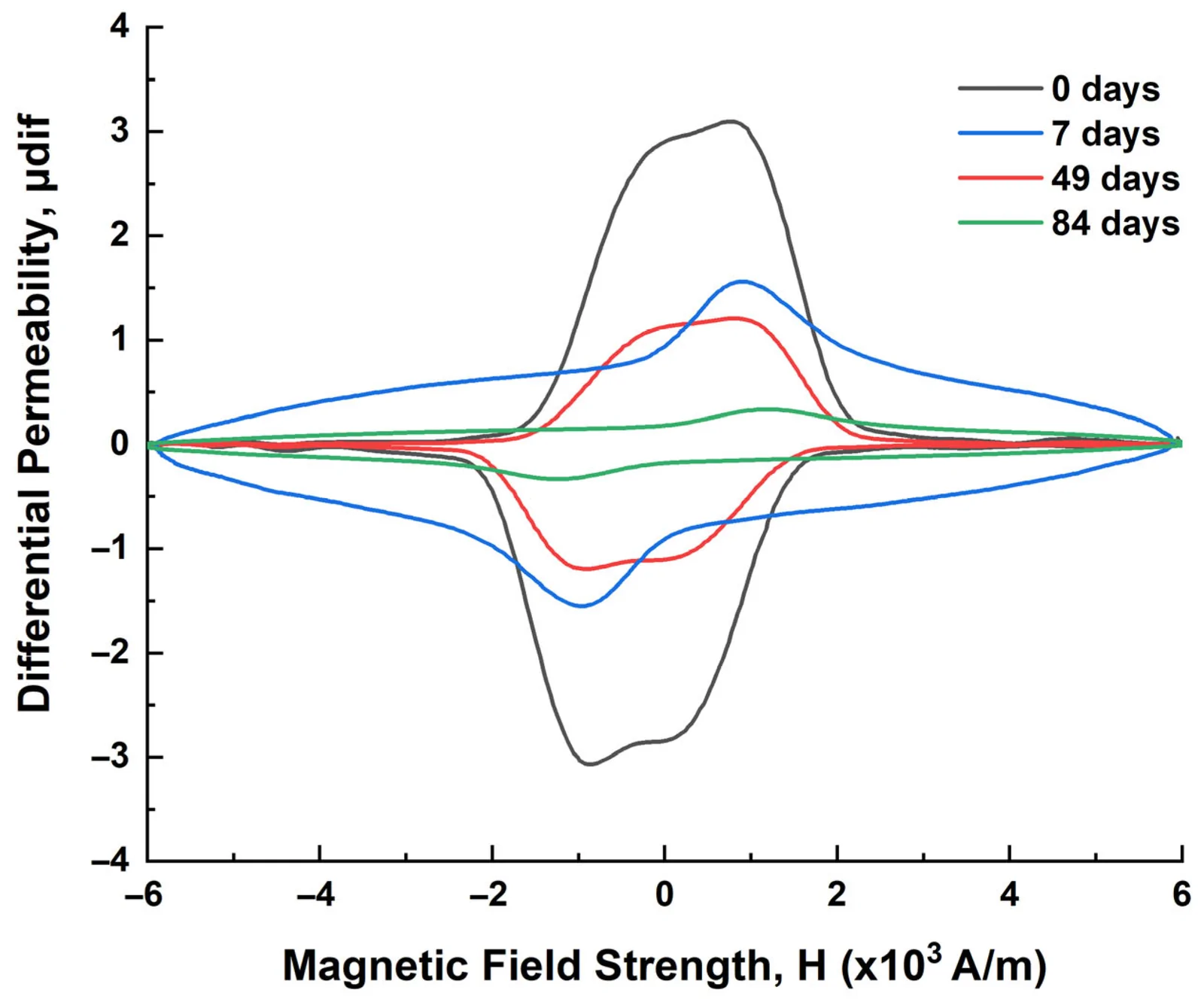
Magnetic permeability loops of the electrochemically corroded naval steels.

The magnetic permeability loops (Figure 5a) of the corroded steel samples were selected for the non-destructive examination of corrosion behavior of immersed naval steels. To facilitate comparability, the corresponding loop of the non-corroded sample was also assessed. Figure 12 displays the results of the measurements.

The formation and evolution of corrosion products on naval steels during immersion in artificial seawater exert profound effects on the magnetic behavior of the underlying metal, particularly on its magnetic permeability. In the early stages of corrosion, products such as lepidocrocite (γ-FeOOH) and goethite (α-FeOOH) (Figure 11a) are paramagnetic or weakly antiferromagnetic and form discontinuous, poorly adherent films [49]. These initial layers provide minimal interruption to the steel’s ferromagnetic matrix, resulting in relatively minor changes in overall magnetic permeability. However, as corrosion progresses and the product layer thickens, the accumulation of non-magnetic or weakly magnetic phases increasingly separates the ferromagnetic bulk from the measurement probe, causing a pronounced decrease in measured permeability [55,56].

Over more extended immersion periods (49 and 84 days), the complexity of the corrosion layer increases, with internal stratification and the formation of additional phases such as maghemite (γ-Fe_2_O_3_), and akaganeite (β-FeOOH) (Figure 11b,c) [57]. Among these, maghemite is ferrimagnetic and can locally enhance magnetic permeability if present in significant quantities and well-adhered to the steel substrate [58]. However, these phases are typically intermixed with the dominant non-magnetic oxyhydroxides, diluting their magnetic contribution. The growing thickness and heterogeneity of the corrosion layer thus tend to lower the net magnetic permeability detected by surface-sensitive techniques, as the eddy currents or magnetic flux must traverse increasing amounts of non- or weakly magnetic material.

The degree to which corrosion products modulate magnetic permeability also depends on their physical characteristics and distribution. Porosity, microcracks, and the presence of occluded rust phases not only reduce the continuity of the ferromagnetic path between the steel and measuring sensor, but may induce localized demagnetizing fields and discontinuities in magnetic flux [55]. As a result, both the amplitude of permeability loops and the sharpness of magnetic hysteresis decline with increasing corrosion duration and layer complexity. Such trends have been confirmed in empirical studies, where magnetic permeability steadily decreases with the accumulation of corrosion products, whether in the form of adherent rust or loosely bound, porous residues.

The values of the magnetic parameters derived from the magnetic permeability loops are presented in Table 7. The variations in the values indicate the sensitivity of these parameters to the annealing conditions.

The configuration of the magnetic differential permeability loops (Figure 11) transitions from a squarer form with heightened vertical development to a more flattened and expanded shape as the residence time in the ASW rises. The greatest value of magnetic permeability (μ_max_) is found in the coercivity field. Magnetic permeability loops do not display secondary peaks until the magnetic field value matches the coercivity field, therefore indicating that no stress is infused from corrosion, as corroborated by magnetic measurements [7]. The reduction in μ_max_ underscores that hysteresis loops, and hence magnetic permeability loops, are assessed along a hard magnetization axis. The presence of corrosion products on the steel surface and the thickening of the corrosion layer result in a decrease in the frequency of irreversible magnetization processes, hence diminishing the maximum magnetic permeability values (μ_max_).

The maximum peak permeability moves to higher magnetic field levels (Hμ_max_) as the immersion duration in the ASW grows. Corrosion products, the weight reduction of the sample, and microstructural imperfections within the corrosion layer impede the magnetization process and result in artificially induced magnetic barriers. This leads to an elevation in the values of Hμ_max_. Nonetheless, over a 49-day duration, the value of Hμ_max_ is observed to be marginally diminished in comparison to the 7-day duration in ASW. This divergence from the typical trend of rising Hμ_max_ values with prolonged immersion time is likely attributable to the more uniform surface profile of the corrosion layer, resulting from the dense morphology of the corrosion products. The lack of significant roughness enhances the efficient interaction between the sensor coils and the underlying naval steel. The presence of magnetic oxides in the corrosion layer likely facilitates the mobility of the magnetic walls.

The expansion of the magnetic permeability loops, as evidenced by the rise in FWHM values, indicates the existence of corrosion-induced stresses and significant structural defects that generate additional pinning centers, hence complicating the movement of the magnetic walls [59]. The values reach their peak on the 84th day of immersion in ASW, as the resistance from the pinning sites to the movement of the magnetic walls greatly increases, along with the ratio of the 180° and 90° magnetic walls implicated in the impeded movement [60,61,62,63].

The H_RS_ values are inferior to the equivalent H_LS_ values, indicating that following magnetic saturation, the samples are more challenging to demagnetize (elevated H_LS_ values) due to corrosion enhancing the isolated ferromagnetic regions, thereby diminishing the continuous ferromagnetic pathways [60]. The motion of magnetic walls and the rotation of magnetic dipoles is facilitated (H_RS_ < H_LS_) during the magnetization process. This trend is reversed with short residence times in ASW, since corrosion rates are elevated and the loss of magnetic material is more significant.

The aforementioned conclusions are further substantiated by the fluctuations in the values of the parameters A1_L_ and A1_R_. The diminished values of A1_R_, relative to A1_L_, suggest the facilitation of the rotation of magnetic dipoles occurring at elevated magnetic fields, in contrast to the displacement of magnetic walls (180°, 90°) that transpires at lower fields [60,61].

**Figure 13:**
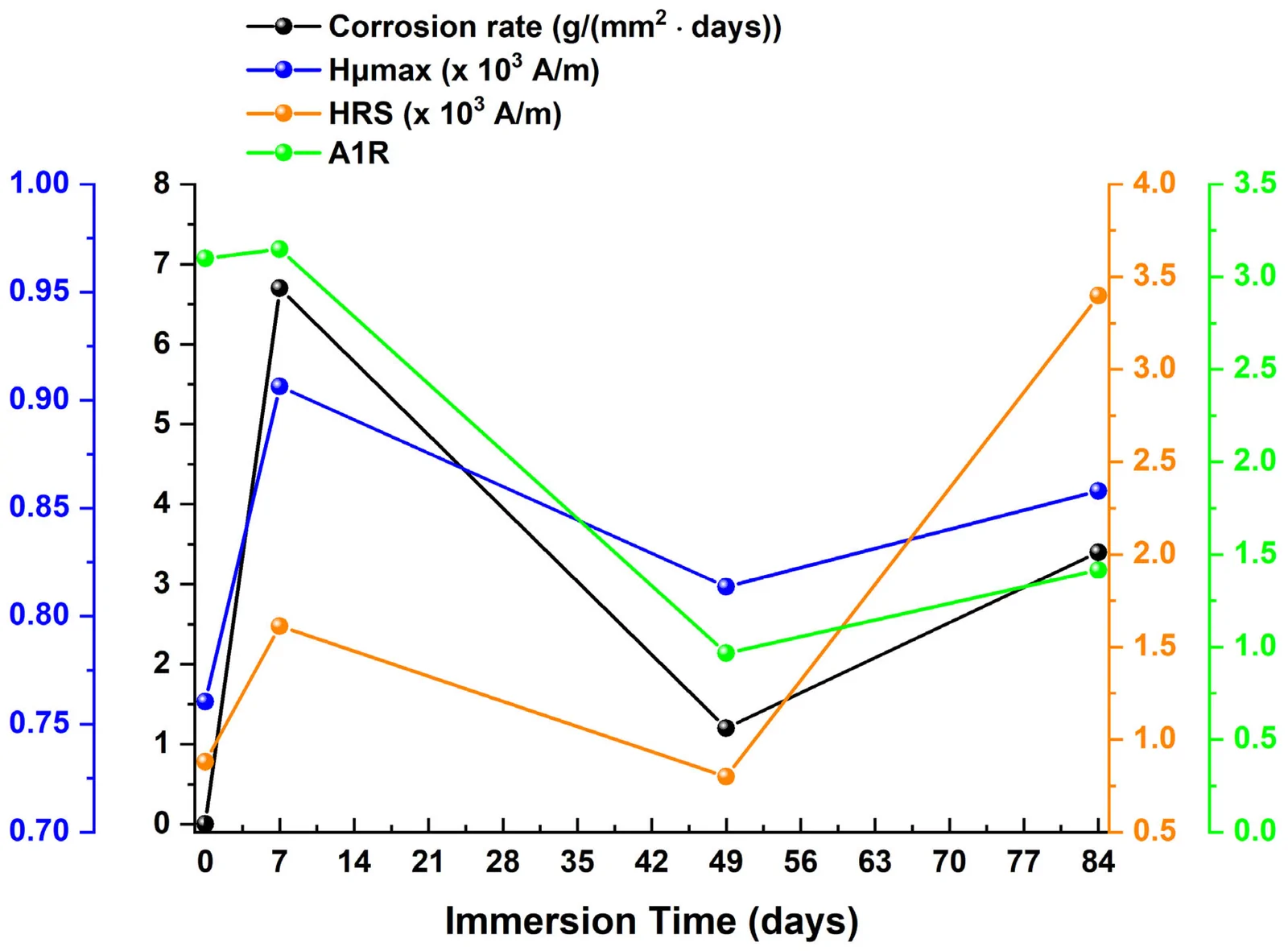
Variation in corrosion rate and magnetic parameters as function of immersion time.

**Figure 14:**
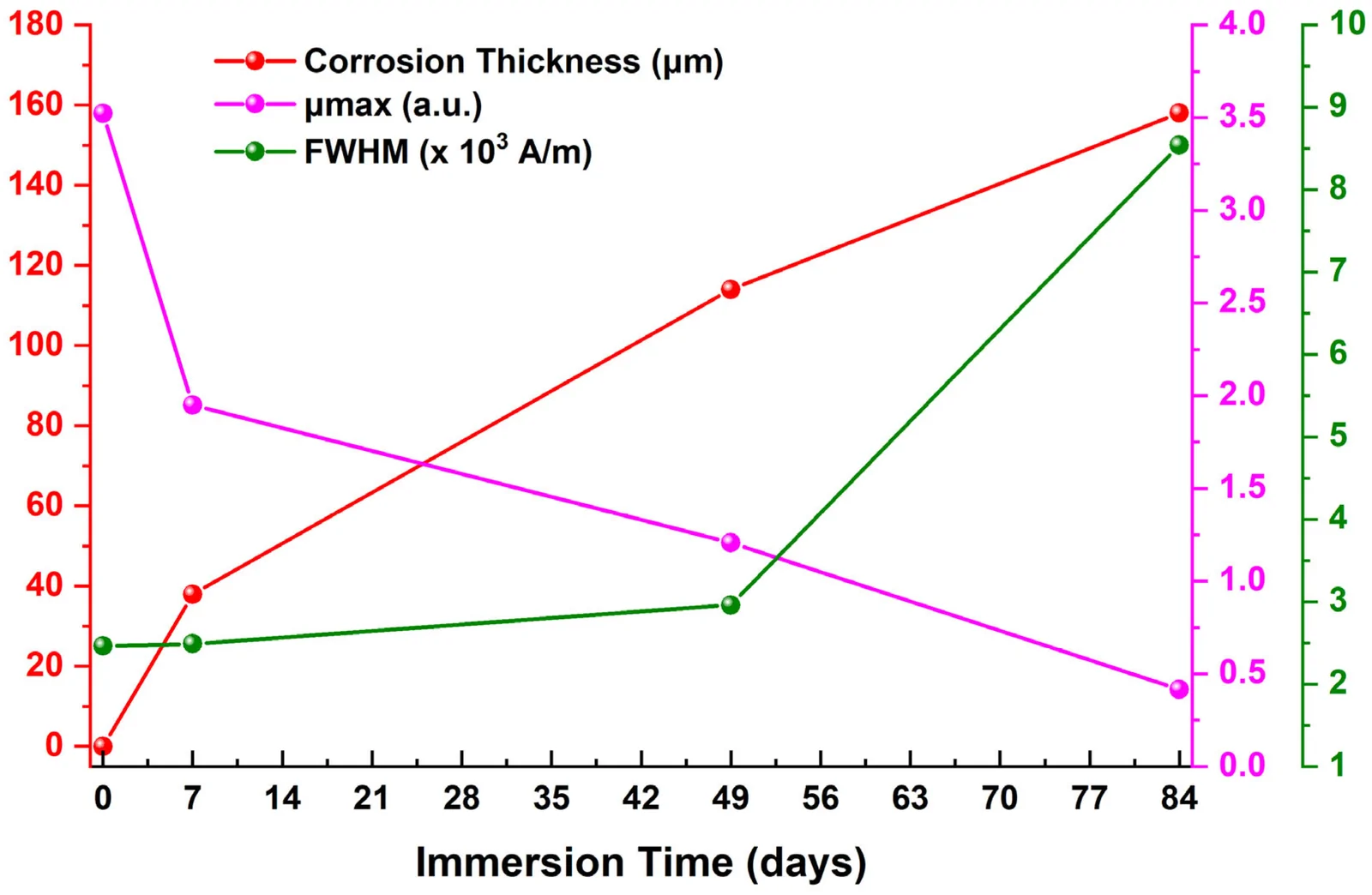
Variation in corrosion layer thickness and magnetic parameters as function of immersion time.

**Figure 15:**
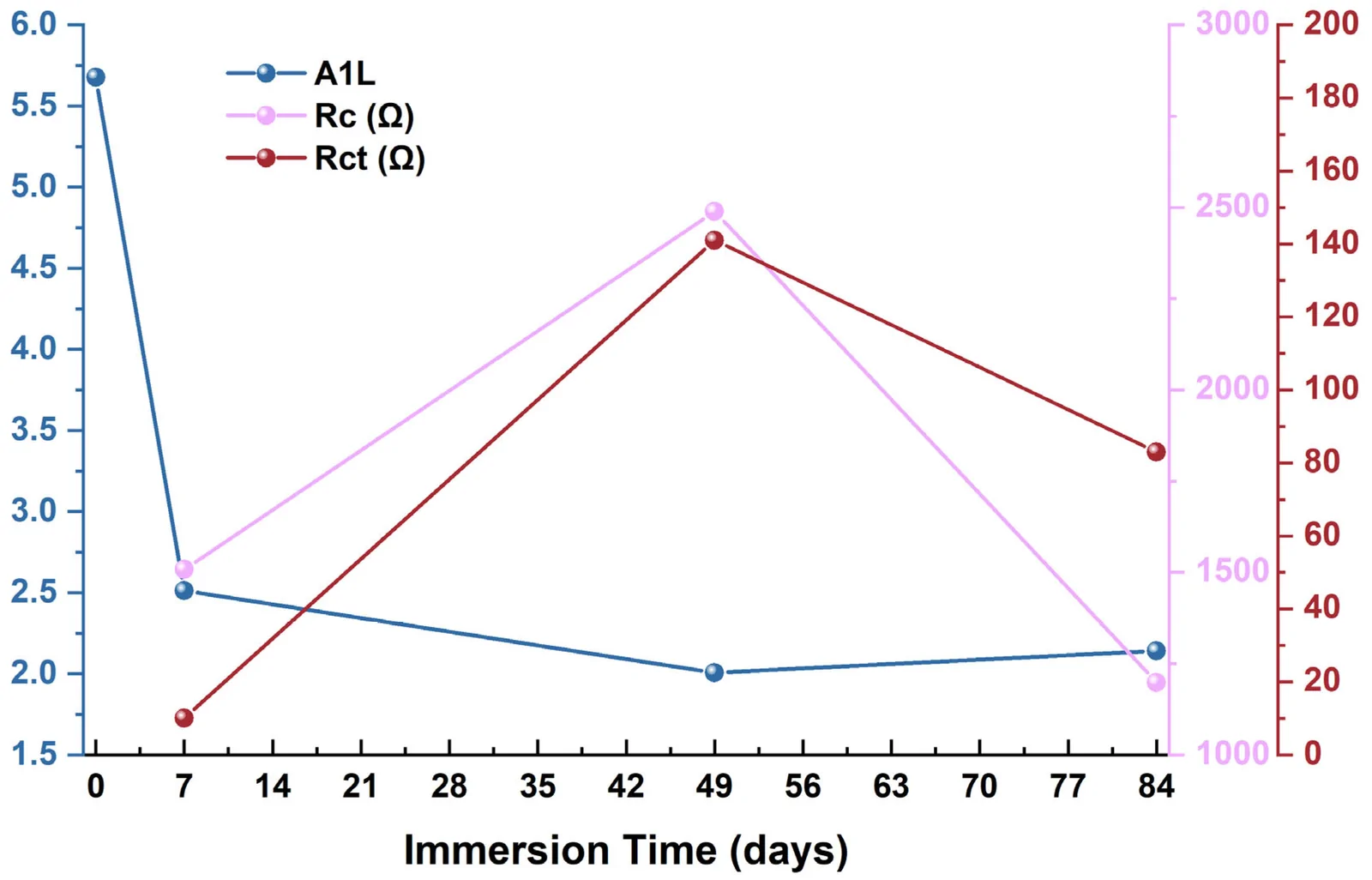
Variation in the resistance’s values of equivalent electric circuit of electrochemical corrosion and magnetic parameters function of immersion time.

**Figure 16:**
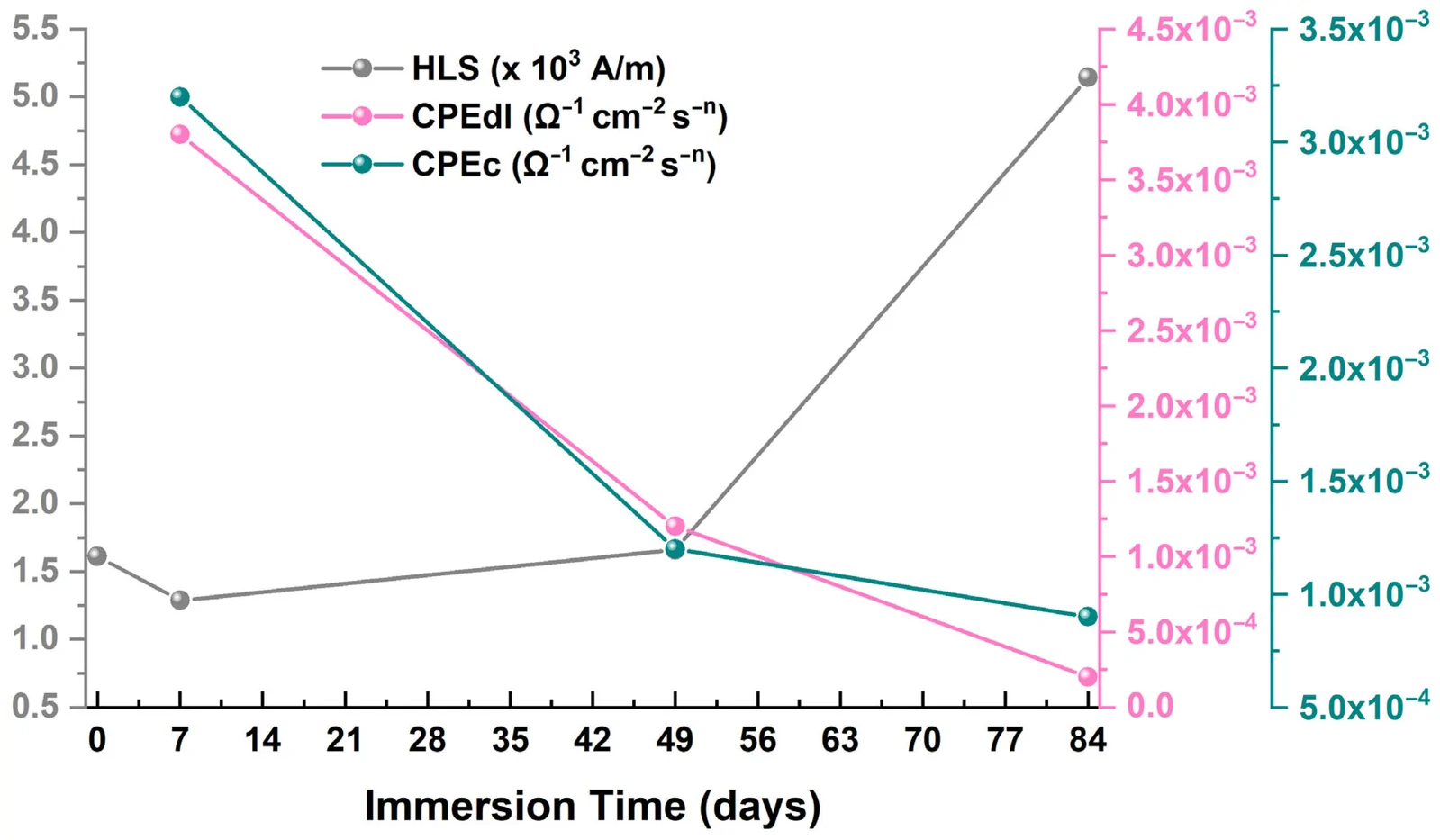
Variation in the capacitance’s values of the equivalent electric circuit of electrochemical corrosion and magnetic parameters function of immersion time.

By examining the fluctuations in magnetic parameters and juxtaposing them with corrosion rate (Figure 13), corrosion thickness (Figure 14), resistance values (Figure 15), and capacitances (Figure 16) of the equivalent electrical circuit of electrochemical corrosion, we discern direct correlations. The corrosion rate exhibits a consistent relationship with Hμ_max_ and H_RS_ values. The sensitivity of the A1_R_ parameter is inadequate for correlating its variation with the corrosion rate. The thickness of the corrosion layer has an inversely proportionate relationship with the highest value of magnetic permeability. The FWHM cannot be directly connected. The resistivity components of the electrical equivalent circuit in Figure 8 exhibit a monotonic change with the A1_L_ parameter, whereas the CPE values demonstrate an inversely proportional relationship with the H_LS_ values.

### 3.2. In Situ Corrosion Tests

**Figure 17:**
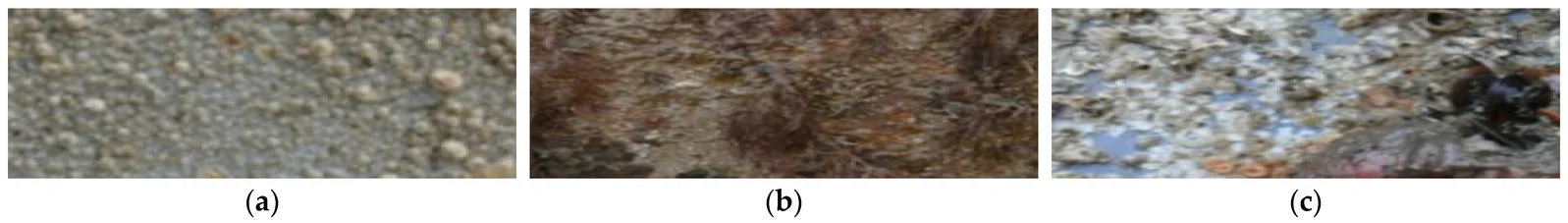
Digital photographs of naval steel samples’ surfaces (2 cm × 6 cm) immersed in natural seawater for (**a**) 7 days, (**b**) 49 days, and (**c**) 84 days.

Naval steel specimens were immersed in the marine environment of Rafina for durations of 7, 49, and 84 days. Figure 17 displays representative digital photos of the samples’ surfaces upon their extraction from natural seawater.

**Figure 18:**
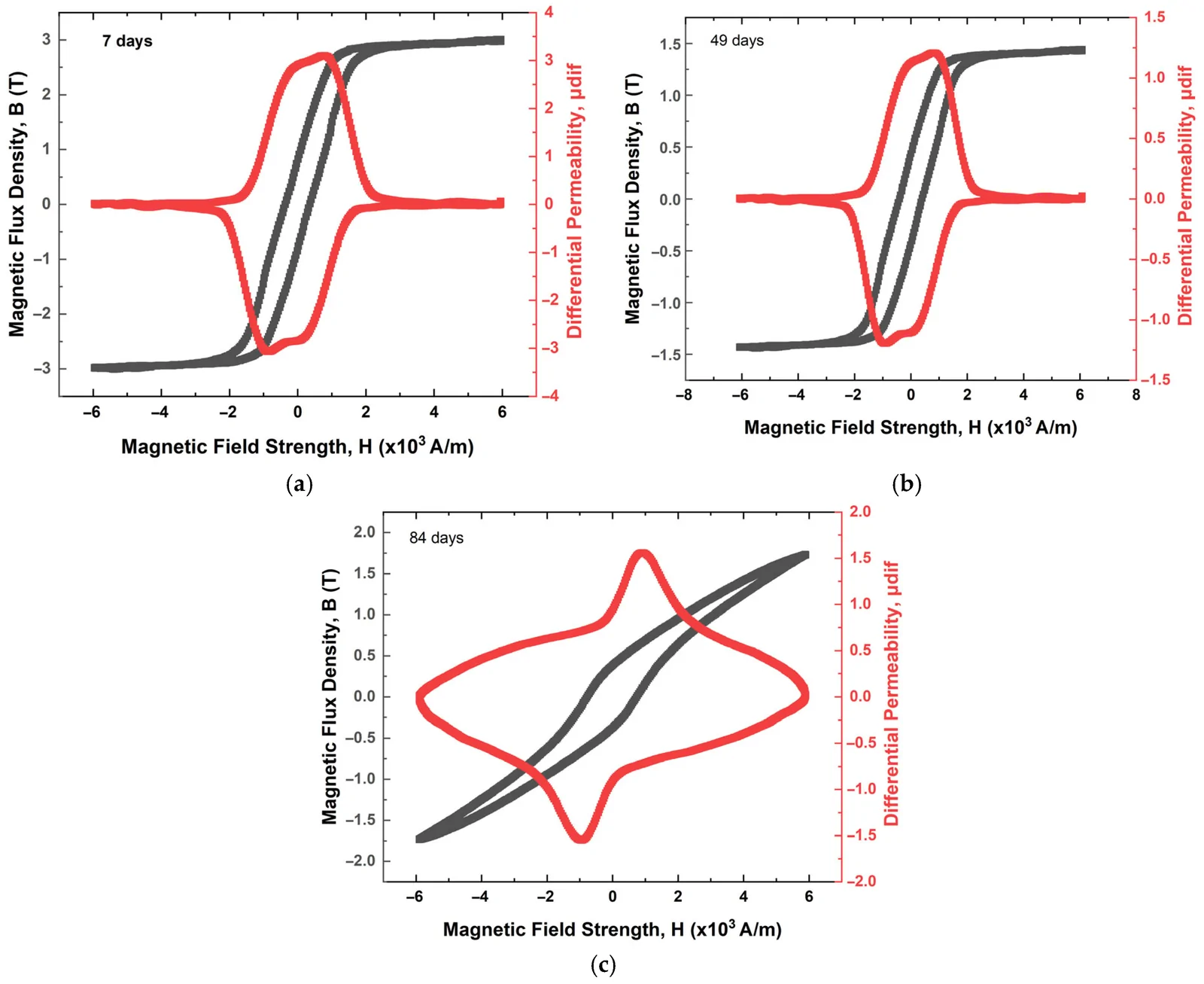
Magnetic hysteresis loops (black) and magnetic permeability loops (red) of naval steel specimens immersed in natural seawater for (**a**) 7 days, (**b**) 49 days, and (**c**) 84 days.

Upon the removal and desiccation of the materials, their magnetic permeability loops were assessed. The results acquired are illustrated in Figure 18.

The presence and maturation of biofilm products (Figure 17) on corroded naval steels substantially impact their magnetic properties, particularly magnetic permeability (Figure 18). Initially, biofilm formation is dominated by the secretion of extracellular polymeric substances (EPS), which create an organic, hydrated barrier atop the steel surface. While these EPS-rich layers are non-magnetic and poorly conductive, their physical separation of the steel from oxygen—and subsequent alteration of electrochemical gradients—indirectly influences the formation and accumulation of corrosion products with distinct magnetic properties. Early-phase corrosion products under biofilm, such as lepidocrocite (γ-FeOOH) and goethite (α-FeOOH), are weakly magnetic or antiferromagnetic, and biofilm coverage may slow their transformation or promote localized environments conducive to alternative iron phases [64,65,66].

As biofilms mature and microbial activity intensifies (Figure 17b,c), especially in marine environments, there is often a shift toward anaerobic conditions at the steel/film interface. Certain microbes, particularly sulfate-reducing bacteria (SRB) and iron-oxidizing bacteria, promote the formation of iron sulfides (e.g., FeS, Fe_3_S_4_ or greigite), which are often more magnetic than typical iron oxyhydroxides but are intermixed with non-magnetic organics and corrosion products [66,67,68]. The complex and stratified microenvironments produced by biofilms thus give rise to highly heterogeneous layers exhibiting both ferrimagnetic (from sulfides or magnetite produced under reducing conditions) and non-magnetic (organic or oxyhydroxide) characteristics. This heterogeneity results in variable, often increased, effective magnetic permeability as measured at the surface.

Magnetic permeability is especially sensitive to both the physical thickness of non-magnetic biofilm layers and to the microstructure of underlying corrosion products. Thick, hydrated biofilms act as barriers to the direct penetration of magnetic fields, further attenuating the measured permeability beyond what would be expected from the corrosion product alone [64,66,69]. Additionally, increased porosity, heterogeneity, and microcrack formation within these combined biofilm–corrosion layers disrupt magnetic flux continuity. Consequently, the presence of robust biofilms correlates with a measurable decline in surface magnetic permeability, often serving as a useful diagnostic signal in non-destructive testing of corroded steel under marine exposure.

Finally, the biofilm’s role in mediating corrosion—by promoting spatially varying redox conditions and facilitating the establishment of localized cathodic and anodic regions—can introduce further spatial variation in underlying steel magnetic domains. This effect, when combined with the non-magnetic organic overlay and the microbially mediated formation of magnetic iron sulfides or oxides, renders the correlation between magnetic measurements and corrosion severity more complex but also potentially more sensitive to early-stage biofouling and corrosion onset [65,66,69]. Ultimately, the monitoring of magnetic permeability changes provides valuable, indirect insights into both the chemical and biological evolution of corroded naval steel surfaces in marine environments.

**Figure 19:**
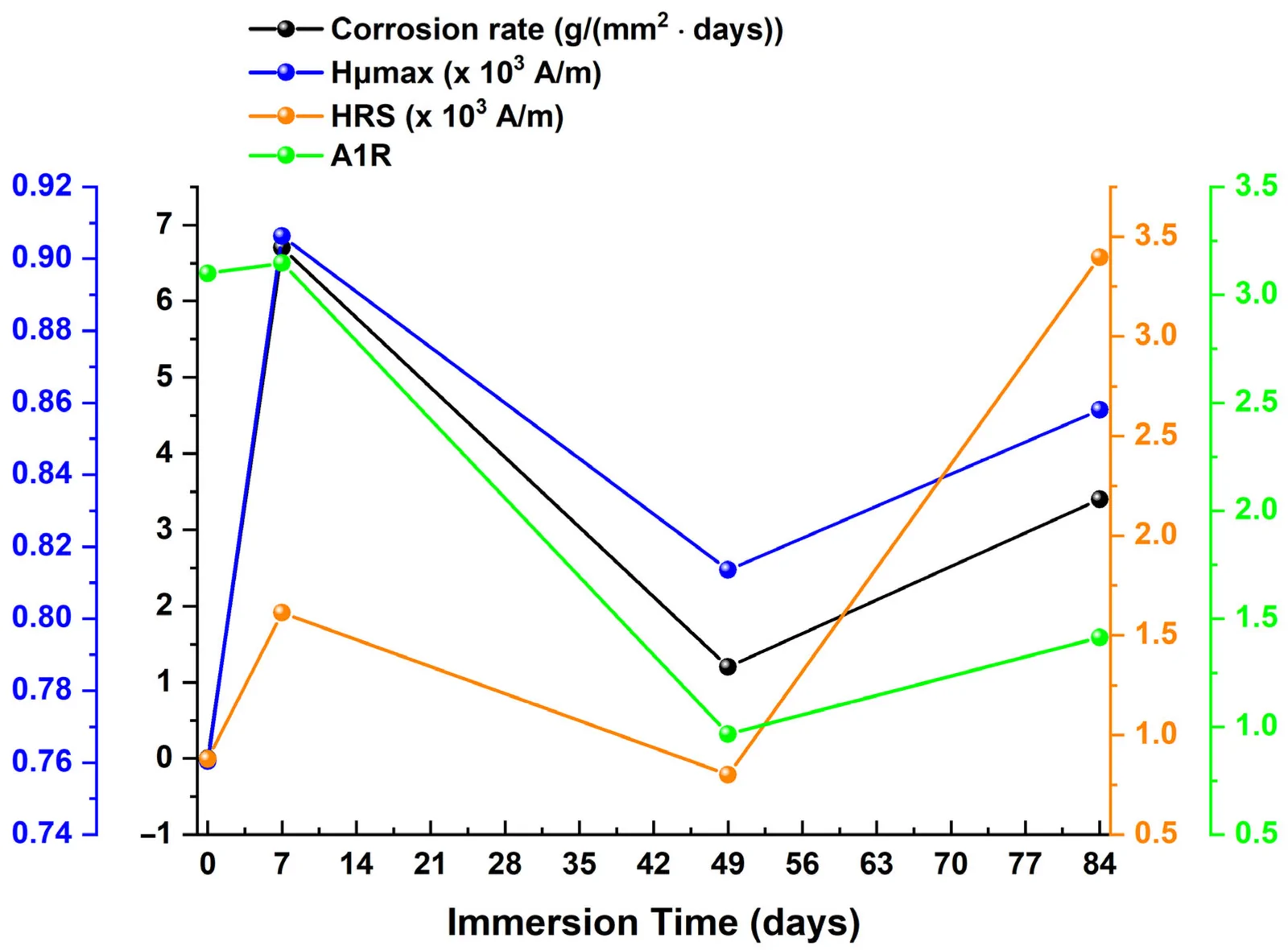
Fluctuation of magnetic properties and corrosion rate as a function of immersion time in natural seawater.

The biofouling layer was removed from the samples, and the corrosion rate was assessed using the weight loss method (Equations (1) and (2)). Figure 19 illustrates the fluctuations in magnetic characteristics and corrosion rate data. The magnetic field value at the peak of magnetic permeability and the area to the right of the intersection of the magnetic hysteresis loop with the magnetic permeability loop exhibit identical monotonic behavior concerning the corrosion rate; however, the variation in H_RS_ values does not correlate well with the corrosion rate.

## 4. Discussion

The present study comprehensively examines the evolution of the corrosion products, corrosion rates, and magnetic properties of naval steel specimens immersed in artificial seawater (ASW) across defined time intervals (7, 49, and 84 days). By integrating electrochemical (EIS), morphological (SEM), and advanced magnetic permeability measurements, the research advances understanding of both chemical and microstructural transformations relevant to marine environments, and positions magnetic non-destructive evaluation as a robust tool for quantifying corrosion progression.

Magnetic permeability is a key characteristic of ferromagnetic materials that indicates the ease with which a material can be magnetized by an external magnetic field [70]. Corrosion modifies the microstructure, composition, and stress state of a material, hence directly influencing its magnetic permeability [2].

When naval steel is unmagnetized, its magnetic domains are randomly orientated, leading to a net magnetization that is non-demagnetized [71]. In magnetic permeability measurements, an external magnetic field is supplied, causing the domains to align with the field, resulting in the growth of some domains at the expense of others, and so enhancing the net magnetization [72].

In the absence of corrosion, the steel exhibits homogenous magnetic domains, distinct grain boundaries, and pronounced imaging contrasts among the magnetic domains. The surface roughness is minimal, and the grains are uniformly oriented and sized. Corrosion results in the formation of holes or fissures on the steel surface. The domain walls are disrupted or uneven, displaying diminished or altered magnetic alignment, which correlates with a decrease in the material’s magnetic response [73,74]. Table 8 encapsulates the impact of corrosion on the magnetization process.

Initial immersion (7 days) results in the rapid formation of loosely adherent, granular corrosion products, primarily consisting of lepidocrocite (γ-FeOOH) and goethite (α-FeOOH), as confirmed by XRD and SEM observations (Figure 9 and Figure 11a). These oxyhydroxide phases are characteristic of the oxygen-rich, chloride-containing ASW environment and are known for their limited protective capability, leaving exposed regions of steel vulnerable to continuing electrochemical attacks [75,76]. The microstructural appearance aligns with the literature, where high initial corrosion rates are attributed to fast metal dissolution and swift nucleation of primary rust phases under marine conditions [77]. With immersion extended to 49 days, the corrosion layer transforms, exhibiting increased density, adherence, and stratification. SEM cross-sections (Figure 9e,f) and phase analysis reveal partial conversion to more stable goethite, with maghemite (Fe_2_O_3_) emerging near the steel interface as oxygen becomes less accessible, in agreement with findings by Xiao et al. [78].

By 84 days, thick, stratified corrosion films are evident, with SEM and XRD (Figure 9c,f and Figure 11c) confirming the presence of akaganeite (β-FeOOH)—a chloride-rich phase—alongside denser goethite and maghemite. Akaganeite’s appearance is directly linked to high chloride ingress and the maturation of occluded rust microenvironments, as noted in Gotoh et al. [79]. The product layer at this stage develops significant porosity and microcracking, underpinning localized corrosion and reactivation phenomena observed in long-term marine immersion studies. Importantly, this evolution of phase composition and internal morphology directly governs the electrochemical and physical properties described below.

The results (Figure 10) document a non-linear corrosion rate profile: an initial spike (up to day 7), a sharp reduction (days 7–49), and a secondary, subdued increase by day 84. This sequence mirrors established multi-phase corrosion models for marine steels, and is substantiated by Vukelic et al.’s long-term exposure studies [47]. The high initial corrosion rate stems from abundant oxygen and clean steel surface, but as corrosion product builds up, inward oxygen diffusion is increasingly impeded; the resulting drop-in corrosion rate (by day 49) corresponds to a more compact, albeit still imperfect, oxide film. The renewed, albeit lower, increase at 84 days reflects breakdowns in film continuity—evidenced by microcracks and increased localized porosity—permitting episodic access of corrosive agents to the underlying metal, a trend further corroborated by Melchers’ anaerobic/microbial acceleration model and phase transitions documented in related works.

The evolving corrosion rate in this work compares well to Vukelic et al.’s findings [48], where initial exposure to natural seawater led to mass loss and high corrosion rates that moderated over time as protective products accumulated, then stabilized around 0.10–0.15 mm/year. Such quantitative congruence affirms the validity of using ASW and laboratory protocols to emulate real-world corrosion mechanisms.

Electrochemical impedance spectroscopy (EIS) data, particularly the Nyquist and Bode plots (Figure 7a–c), display multiple depressed semicircular arcs, most notably at extended immersion times, indicating more than one relaxation process. This necessitated the use of a multi-time-constant equivalent circuit (Figure 8), comprising distinct R_C_–CPE branches for both corrosion film and double layer. This is in line with advanced interpretations in the field that emphasize the inadequacy of simple Randle’s circuits for layered corrosion systems. Parameter values extracted (Table 5) show increasing corrosion layer resistance and decreasing CPE with prolonged exposure, confirming rust densification and the transition to diffusion-controlled kinetics.

Magnetic permeability measurements, visualized through permeability loops and related parameters (Figure 5, Figure 14 and Figure 18), show progressive declines in peak permeability (μ_max_) with increasing corrosion product thickness and exposure duration (Table 6, Figure 14). These observations reflect the accumulation of weakly magnetic (lepidocrocite, goethite) or non-magnetic (porous rust, biofilm) phases, whose presence reduces the net ferromagnetic path between the steel and the probe. Only in localized environments where maghemite are dominant can small, transient increases in permeability occur.

Other parameters—such as coercive field, full width at half maximum (FWHM), and area under the BH loop—also track with microstructural changes, surface roughness, and crack morphology, consistent with mechanistic proposals of domain wall pinning under corrosion and magnetic non-destructive evaluation literature [80]. The congruence of magnetic signatures with physical corrosion layer metrics substantiates the application of magnetic permeability analysis for corrosion monitoring, supplementing traditional weight loss and electrochemical approaches.

More specifically, in the early phases of corrosion (0–7 days), magnetic naval steel begins to exhibit non-magnetic iron oxides or hydroxides on its surface as corrosion products (Figure 9a), which possess significantly lower magnetic permeability than steel [2]. As a result, the magnetic permeability of the material diminishes (Table 6). To finalize the magnetization, elevated magnetic fields are necessary to orient the magnetic dipoles (increase of Hμ_max_), resulting in an extended duration for the magnetization process (increase in FWHM). The magnetic permeability loops lack symmetry, resulting in unequal values of H_RS_ and H_LS_. A greater H_RS_ value signifies the full rotation of magnetic dipoles at elevated fields, attributed to the challenges in magnetizing the material caused by the presence of corrosion products. The elevated corrosion rate resulting from the swift diffusion of iron oxide generated at the anode in seawater necessitates heightened energy demands to finalize the magnetization of the material (increase in A1_R_). The reduction in steel weight and the subsequent rise in corrosion rate are substantiated by the CPE values in the electrical equivalent circuit. Elevated CPE values signify the existence of ions at the steel contact of a nascent corrosion layer.

As the corrosion reaction advances and a solid layer of corrosion products forms on the steel surface, structural flaws (pits, voids, and microcracks) are created, disrupting the alignment of the magnetic domains when a magnetic field is applied. This therefore results in the suppression of the material’s magnetism, rendering the naval steel less capable of conducting magnetic flux, particularly when a substantial layer of corrosion has developed (49 days of immersion). This compact layer, characterized by minimal porosity, serves as a barrier at the steel interface, inhibiting corrosion through diminished CPE values and a reduction in CR. The corrosion layer exhibits minimal surface inhomogeneity, resulting in a more symmetrical magnetic circuit of the magnetic potential lines and a reduction in Hμ_max_. The existence of a layer of non-magnetic corrosion products inhibits the magnetization of the material by elevating the magnetic parameter FWHM. The mobility of the magnetic walls is more impeded during the demagnetization of the material post-saturation, as indicated by elevated values of H_LS_ and A1_L_ compared to the comparable H_RS_ and A1_R_.

As corrosion advances, the continuity of the magnetic pathway diminishes alongside a loss in steel thickness, leading to a further decline in magnetic permeability (84 days). In this instance, despite the increasing thickness of corrosion, the significant presence of structural flaws, primarily fractures (resulting in a decrease in CPE and R in the electrical equivalent circuit), leads to a subsequent rise in the corrosion rate alongside the overall magnetic parameters.

Table 9 demonstrates the progression of the parameter values presented in Table 6 and Table 7. The upward arrow indicates an increasing trend, whereas the downward arrow represents a decreasing value at the corresponding immersion time.

The corrosion rate exhibits a monotone behavior with the Hμ_max_ and H_RS_ parameters in both electrochemical corrosion (Figure 13) and actual immersion circumstances (Figure 19). The ability of magnetic permeability to evaluate corrosion levels in ferromagnetic materials was investigated in actively corrosive settings, specifically under static immersion conditions in seawater. In very aggressive maritime conditions, where steel corrosion is accompanied by a substantial coating of biofouling, magnetic permeability exhibits a monotonic relationship with the corrosion rate.

**Figure 20:**
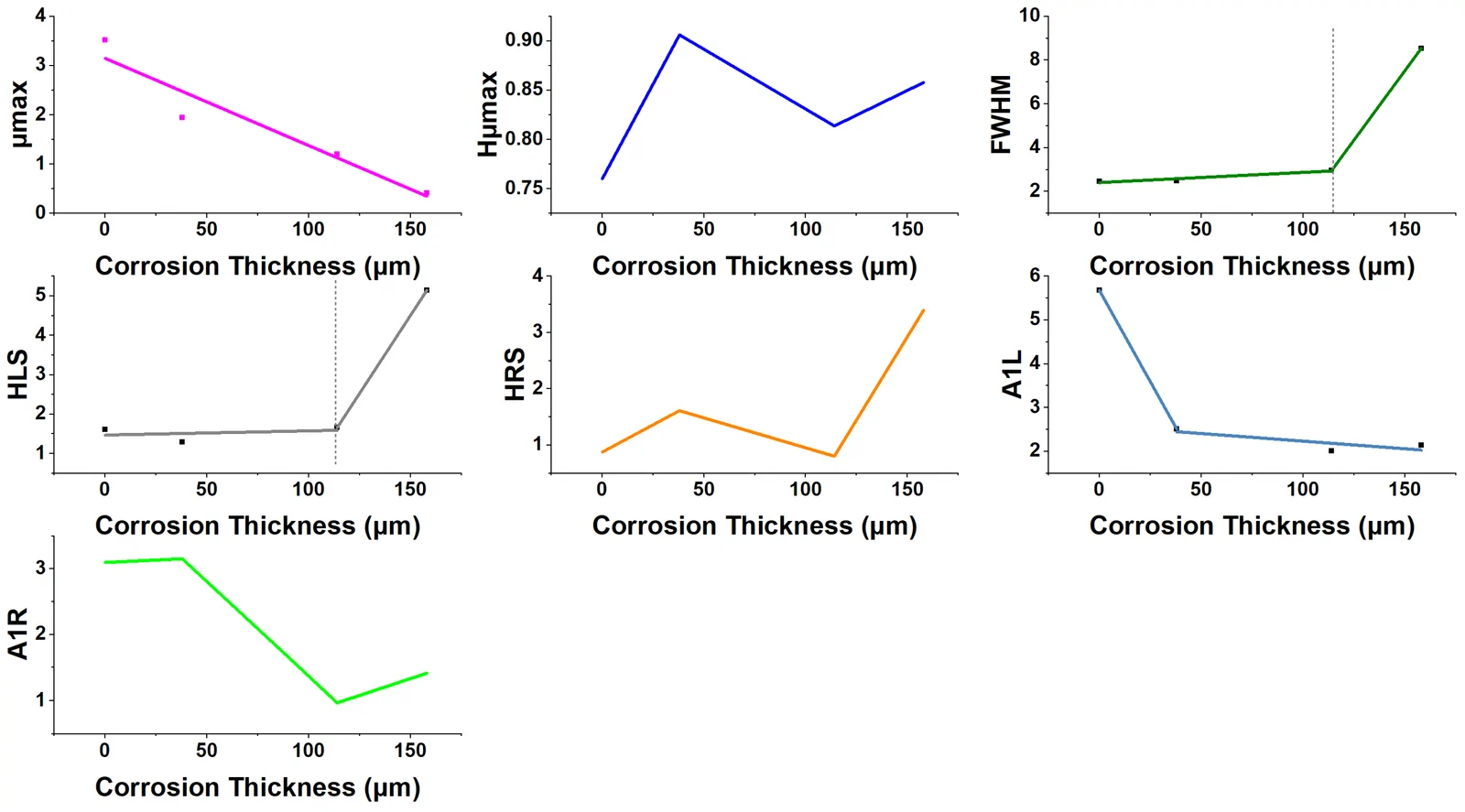
Relationship between corrosion layer thickness and magnetic parameters for the naval steel samples corroded in ASW.

Among the magnetic parameters, μ_max_ has an inverse proportionality to the thickness of the corrosion products for the naval steel samples corroded in ASW. In Figure 20, the FWHM and H_LS_ parameters exhibit measurement sensitivity for corrosion thicknesses exceeding 120 μm; additional data points are required to ascertain whether a significant inflection occurs at a specific corrosion thickness. The A_1L_ measure exhibits a comparable tendency for thicknesses up to 45 μm; however, the sensitivity in assessing corrosion thicknesses is still under examination. The remaining magnetic properties cannot be associated with the thickness of the corrosion layer.

A review of relevant corrosion and detection studies underscores the novelty and reliability of the methods and interpretations herein. Table 10 summarizes key works, materials, test conditions, and findings.

This comparative framework affirms the generalizability—and quantitative soundness—of the results, and highlights the present study’s unique focus on correlating corrosion products with magnetic permeability for predictive, non-destructive monitoring in marine steels.

The results confirm that corrosion and magnetic permeability of naval steels are directly, quantifiably linked via the progressive, stratified build-up of non-magnetic corrosion products. Such linkages provide a mechanistic basis for rapid, non-contact assessment of corrosion state in marine infrastructure, facilitating the more sensitive detection of early-stage attack and supporting condition-based maintenance. Furthermore, the use of EIS and carefully selected equivalent circuits enhances the extractable electrochemical insights, especially for multilayered, real-world corrosion films.

Nevertheless, several limitations warrant mention. First, while artificial seawater conforms well to ASTM D1141 and suitably accelerates certain processes, the true complexity of natural marine environments—including variable flow rates, biofouling, temperature, and microbial activity—may not be fully replicated. Second, the focus on DH36 steel, though representative, may limit the extension of quantitative results to other steel chemistries or treatments; future work should broaden compositional and environmental scope. Lastly, while the experimental coincidence of corrosion rate, morphology, and magnetic property trends are robust, further analytical cross-validation (e.g., synchrotron XRD, nanoindentation, high-resolution EDX) would enrich mechanistic details, especially concerning minor corrosion phases and nanoscale magnetic effects.

In conclusion, corrosion progression in naval steels immersed in artificial seawater closely follows a sequence of microstructural, chemical, and magnetic changes that can be quantitatively monitored by combining EIS, morphological, and magnetic techniques. The present findings substantiate the multi-phase corrosion model, affirm prior immersion studies, and uniquely integrate magnetic permeability mapping as a sensitive, non-destructive indicator of corrosion state. The results support predictive maintenance approaches and establish a path toward high-throughput, real-time corrosion sensing systems for marine platforms.

## 5. Conclusions

This study provides a thorough investigation into the temporal evolution of corrosion and its effects on the physical, electrochemical, and magnetic properties of naval steel immersed in artificial seawater. By employing a combination of weight loss measurements, electrochemical impedance spectroscopy (EIS), scanning electron microscopy (SEM), and non-destructive magnetic permeability analysis, the research elucidates both the kinetic and mechanistic aspects of corrosion development across multiple time scales.

The results clearly demonstrate that the corrosion of DH36 naval steel in artificial seawater progresses through distinct morphological and electrochemical phases. Initial exposure is marked by rapid metal dissolution and the formation of loosely adherent lepidocrocite and goethite, which afford limited barrier protection. Prolonged immersion leads to the development of compact and stratified corrosion layers comprising goethite, maghemite, and akaganeite, accompanied by the onset of microcracking and increased porosity. These microstructural transformations are directly reflected in the non-linear trends of corrosion rate: a prominent initial increase, sharp reduction as the protective layer forms, and a later-stage resurgence associated with localized breakdown and under-film attack.

EIS measurements, validated using two-time-constant equivalent electrical circuits, accurately capture the emergence of multiple overlapping relaxation processes attributable to the layered nature of corrosion product films. The experimental impedance data affirm that the multilayered corrosion system cannot be adequately described by simpler circuits, highlighting the need for advanced modeling to resolve the contributions of the corrosion product and double-layer interfaces.

Magnetic characterization further reveals that the accumulation of non-magnetic (or weakly magnetic) corrosion products induces a measurable and progressive decline in magnetic permeability. Parameters such as peak magnetic permeability, coercivity, and hysteresis width are shown to correlate sensitively with the thickness and morphology of the corrosion products, supporting the utility of magnetic methods as rapid, non-destructive corrosion diagnostics. Combined with SEM imaging and phase identification, these results enable a holistic understanding of the relationships between corrosion kinetics, electrochemical phenomena, and emergent magnetic behaviors.

Comparative analysis with the existing literature demonstrates that the trends identified herein—for corrosion rate evolution, phase sequence, and magnetic response—are robust and representative of marine-exposed steels in both laboratory and natural environments. The integration of electrochemical, morphological, and magnetic data provides a comprehensive framework for real-time corrosion monitoring and service-life prediction in marine infrastructure.

Looking forward, this approach not only substantiates the use of artificial seawater and multi-modal analysis for accelerated corrosion assessment, but also underscores the potential for deploying magnetic non-destructive evaluation for in situ monitoring of naval steels. Future studies should expand to diverse steel chemistries and real-world marine environments, incorporating additional surface and microanalytical techniques to resolve nanoscale features and the role of biofouling or microbial action.

In summary, the findings of this work significantly advance the mechanistic and practical understanding of corrosion in naval steels, bridging microstructural evolution and functional properties. The correlations demonstrated between corrosion progression and magnetic signatures pave the way for the development of smarter, condition-based maintenance strategies vital for the longevity and safety of marine assets.

## Figures and Tables

**Table 1:** Comparison of corrosion protection methods for naval steels.

Protection Method	Principle	Advantages	Limitations	Typical Applications	Refs.
Protective Coatings (Paint, Epoxy, Polymeric)	Creates a physical barrier between steel and environment	Cost-effective, easy to apply, customizable, wide usage	Requires regular maintenance; damaged areas expose steel; UV/salt degrade coatings over time	Ship hulls, superstructures, tanks	[5,6,7]
Cathodic Protection (Sacrificial Anode/Impressed Current)	Converts steel into the cathode uses sacrificial metals (e.g., zinc, aluminum) or electric currents	Highly effective for submerged/underground parts; provides continuous protection	Sacrificial anodes must be replaced; impressed current systems are costly and require monitoring	Ship hulls, ballast tanks, offshore platforms	[4,8]
Galvanizing (Hot-dip Zinc Coating)	Steel is dipped in molten zinc layer protects via barrier and sacrificial action	Long-lasting; good for atmospheric/marine exposure; relatively low cost	Surface prep critical; coating can be damaged; limited to certain shapes/sizes	Fasteners, pipework, structural steel, smaller components	[9,10]
Powder Coating	Thermoset/thermoplastic powder fused to surface, forming thick barrier	Thick coverage, durable, environmentally friendly, esthetic options	Requires careful prep, large parts may be challenging; surface damage exposes steel	Hardware, pipelines, deck fittings	[11]
Metallizing (Thermal Spray Al/Zn Alloy)	Sprayed molten metal forms protective metallic barrier	Suitable for complex shapes; long life (10–20 years with maintenance); effective both above and below water	Requires expert application/sealing; may need combined painting; expensive for large areas	Valves, pumps, structural members, decks	[12]
Petrolatum Tape/Cover System	Wraps surface in petroleum-based tape/lining plus cover (e.g., titanium)	Excellent for joints/fittings; impact/chemical-resistant (with titanium covers); easy maintenance	Limited to specific shapes/applications; can be costly for large uses	Pipe joints, pilings, pier substructures	[13,14]
Corrosion-resistant Steel Alloys	Steel alloyed with nickel, copper, phosphorous, etc., to improve resistance	Inherent protection, less maintenance needed	Expensive; not always effective for severe marine conditions	Specialized naval hulls, hard-to-maintain locations	[14]

**Table 2:** Comparison of corrosion detection methods for steel.

Detection Method	Principle	Advantages	Disadvantages	Typical Applications	Refs.
Visual Inspection	Surface observation for discoloration, pitting, or rust	Low cost, simple, immediate results	Subjective—misses subsurface/internal defects	Routine maintenance, initial screening	[16]
Ultrasonic Thickness Measurement (UTM)	Measures change in material thickness from sound wave reflection	Non-destructive, detects internal thinning	Requires contact and calibration, cannot detect early surface corrosion	Ship hulls, pipelines, structural steel	[17]
Electrochemical Impedance Spectroscopy (EIS)	Evaluates electrochemical response to AC signals to estimate corrosion processes	Sensitive, provides kinetic/mechanistic info	Needs electrolyte contact, limited for in situ/large surfaces	Laboratory monitoring, coating evaluation	
Electrical Resistance (ER) Probes	Monitors changes in electrical resistance as metal loss occurs	Real-time, good for atmospheric and soil environments	Affected by local environment, only area-specific	Storage tanks, soil/pipeline monitoring	
Acoustic Emission (AE)	Detects transient sound waves from active corrosion and cracking	Detects dynamic/hidden/flaw activity, covers large areas	Noisy environments, needs expert data interpretation	Large infrastructure, pressurized vessels	[18]
Magnetic/Eddy Current Testing	Induces electromagnetic field detects anomalies from corrosion/pitting	Good for non-contact, rapid, through-coating inspection	Limited depth, interpretation affected by geometry	Pipelines, coated surfaces, welds	[19]
Computer Vision/Deep Learning	Automated corrosion detection by analyzing digital images	High-throughput, automates inspection, real-time possible	Needs large, labeled datasets, sensitive to lighting conditions	Automated monitoring, asset integrity	[16,20]

**Table 3:** The typical chemical composition of commercial naval steel, expressed as a weight percentage (%wt).

Elements	Fe	Mn	Si	C	P	S
%wt	base	1.23	0.32	0.17	0.025	0.1

**Table 4:** Major constituents per liter of ASW solution, according to the ASTM D1141 standard [23].

Compound	Formula	Amount (g/L)
Sodium chloride	NaCl	24.53
Magnesium chloride	MgCl_2_	5.20
Sodium sulfate	Na_2_SO_4_	4.09
Calcium chloride	CaCl_2_	1.16
Potassium chloride	KCl	0.695
Sodium bicarbonate	NaHCO_3_	0.201
Potassium bromide	KBr	0.101
Boric acid	H_3_BO_3_	0.027
Strontium chloride	SrCl_2_·6H_2_O	0.025
Sodium fluoride	NaF	0.003

**Table 5:** Frequency ranges in EIS diagrams.

Frequency Range	Frequency Band	Correlated with
10^5^ Hz to 10^3^ Hz	High	resistance of the electrolyte
10^3^ Hz to 10^2^ Hz	Middle	double layer formation
10^2^ Hz to 10^−1^ Hz	Low	steel/electrolyte interface reactions

**Table 6:** Fitting results for coated and uncoated steel in ASW solution.

Immersion Time (Days)	R_S_ (Ω)	R_c_ (Ω)	CPE_c_ (Ω^−1^ × cm^−2^ × s^n^)	n_c_	R_ct_ (Ω)	CPE_dl_ (Ω^−1^ × cm^−2^ × s^n^)	n_dl_
0	8	-	-	-	8	9 × 10^−4^	-
7	10	1508	32 × 10^−4^	0.60	10	38 × 10^−4^	0.87
49	23	2489	12 × 10^−4^	0.75	141	12 × 10^−4^	0.93
84	11	1198	9 × 10^−4^	0.66	83	2 × 10^−4^	0.95

**Table 7:** Magnetic parameters of the corroded naval steel samples.

Immersion Time (Days)	μ_max_ ^1^ (a.u.)	Hμ_max_ ^2^ (×10^3^ A/m)	FWHM ^3^ (×10^3^ A/m)	H_LS_ ^4^ (×10^3^ A/m)	H_RS_ ^5^ (×10^3^ A/m)	A1_L_ ^6^	A1_R_ ^7^
0	3.52362	0.76044	2.46127	1.61173	0.87955	5.67912	3.09920
7	1.95067	0.90634	2.49008	1.28709	1.61372	2.51069	3.14784
49	1.20789	0.81358	2.96043	1.66015	0.80028	2.00528	0.96665
84	0.41621	0.85803	8.53979	5.14238	3.39741	2.14031	1.41404

^1^ peak height; ^2^ peak position; ^3^ full width at half peak height; ^4^ position of right shoulder; ^5^ position of left shoulder; ^6^ area under the BH curve to the left of the intersection (A1_L_); ^7^ area under the BH curve to the right of the intersection (A1_Ρ_).

**Table 8:** Effect of corrosion on magnetic domains and magnetization.

Effect of Corrosion	Impact on Magnetic Domains	Impact on Magnetization
Surface roughness	Domain walls encounter more pinning sites	Reduced magnetic permeability and remanence, lower saturation
Micro-cracking/pitting	Domains become smaller or fragmented	Narrower hysteresis loop
Chemical changes	Disruptions in ferromagnetic structure	Reduced magnetic response overall
Oxidation	Non-magnetic oxides form, reducing domain volume	Decrease in total magnetization

**Table 9:** Enumeration of examined magnetic parameters and their progression in corrosion of naval steel specimens at varying immersion durations in ASW. The downward arrow signifies a reduction in the size value, whilst the upward arrow denotes an increase in the equivalent value.

Immersion Time (Days)	μ_max_	Hμ_max_	FWHM	H_LS_	H_RS_	A1_L_	A1_R_	CR	Corrosion Thickness	Rc	CPEc	R_ct_	CPE_dl_
0–7	↓	↑	↑	↓	↑	↓	↑	↑	↑	↑	↑	↑	↑
7–49	↓	↓	↑	↑	↓	↓	↓	↓	↑	↑	↓	↑	↓
49–84	↓	↑	↑	↑	↑	↑	↑	↑	↑	↓	↓	↓	↓

**Table 10:** Comparative analysis with prior literature.

Work	Steel Type	Medium	Detection Methods	Key Findings	Refs.
Barsoukov and Macdonald	-	-	EIS, Modeling	Multi-RC-CPE circuits for complex corrosion	[37]
Rémazeilles et al.	Iron/Steels	Lab/Museum	XRD, Raman, SEM	Akaganeite/phase evolution in chloride	[54]
Xiao et al.	Carbon steel	Marine atmosphere	Rust/surface XRD SEM	Stratified corrosion, phase sequence	[78]
Gotoh et al.	Fe	Air/Marine	Magnetic permeability	Rust layer reduces measured permeability	[79]
Melchers and Jeffrey	Mild Steel	Marine/SW	Weight loss, modeling	Reaction + diffusion + microbial phases, reacceleration	[51]
Vukelic et al.	AH36	Natural seawater	Weight loss, SEM	Non-linear corrosion, stabilization ~0.12mm/y	[47]
Present Study	DH36	ASW (ASTM D1141)	EIS, SEM, Magnetic	Multi-phase corrosion, declining permeability	-

## Data Availability

The data presented in this study are available on request from the corresponding author. (The data are not publicly available due to privacy.)

## Abbreviations

The following abbreviations are used in this manuscript:

ASW	artificial seawater
EIS	Electrochemical Impedance Spectroscopy
RE	Reference Electrode
CE	Counter Electrode
WE	Working Electrode
OCP	Open Circuit Potential
WL	weight loss ratio
w_i_	weight prior to corrosion
w_f_	weight subsequent to corrosion
CR	Corrosion Rate
A	surface area of the specimen experiencing corrosion
t	time of the corrosion process
SEM	Scanning Electron Microscopy
μ_diff_-H	permeability profile
*R*_s_	electrolyte resistance
*R*_ct_	charge transfer resistance
R_c_	corrosion layer resistance
CPE_dl_	constant-phase element of double layer
*CPE*_c_	corrosion layer capacitance
BH	Magnetic hysteresis loop
μ_max_	Peak height
Hμ_max_	Peak Position
FWHM	Full Width at half peak height
H_LS_	Position of right shoulder
H_RS_	Position of left shoulder
A1_L_	Area under the BH curve to the left of the intersection
A1_Ρ_	Area under the BH curve to the right of the intersection
